# Spatial patterns and temporal trends of tracheal, bronchus, and lung cancer attributed to particulate matter pollution in Asia and its 34 countries and territories, 1990–2021

**DOI:** 10.3389/fpubh.2025.1602454

**Published:** 2025-07-24

**Authors:** Minxia Yang, Feng Xuan, Liejiong Wang, Ying Lou, Shengjian Yu

**Affiliations:** ^1^Department of Radiology, Shaoxing People’s Hospital, Shaoxing, Zhejiang, China; ^2^Department of Radiation Oncology, Zhuji Affiliated Hospital of Wenzhou Medical University, Shaoxing, Zhejiang, China; ^3^Department of Medical Oncology, Zhuji Affiliated Hospital of Wenzhou Medical University, Shaoxing, Zhejiang, China

**Keywords:** lung cancer, ambient particulate matter pollution, household air pollution, Asia, joinpoint regression model, global burden of disease, disability-adjusted life years

## Abstract

**Background:**

This study aimed to investigate the spatial and temporal variations of tracheal, bronchus, and lung cancer (TBLC) attributable to particulate matter pollution across 34 Asian countries and territories from 1990 to 2021.

**Methods:**

Disability-adjusted life years (DALYs) linked to ambient particulate-matter pollution (APMP) and household air pollution (HAP) were obtained from the Global Burden of Disease (GBD) 2021 dataset, and performed analyses stratified by location, gender, and age. Trends in age-standardized DALY rates (ASDRs) were quantified with Joinpoint regression. Decomposition analysis was applied to identify the contributions of population aging, population growth, and epidemiological changes to the alterations in DALYs.

**Results:**

From 1990 to 2021, APMP-related ASDR increased significantly in Asia, while HAP-related ASDR declined. In 2021, East Asia recorded the greatest APMP-associated DALYs across five GBD regions, while both South Asia and East Asia bore the heaviest HAP-related burden. China reported the highest absolute DALYs for each pollutant. Population aging and growth accounted for most of the increase in APMP-related DALYs in Asia, whereas epidemiological change chiefly explained the reduction in HAP-related DALYs. ASDRs were consistently higher in males, with wider sex disparities for APMP-related ASDR. Middle-aged and older adult populations were the most vulnerable age groups.

**Conclusion:**

Although TBLC burden attributable to HAP has fallen, disease linked to APMP remains a pressing public-health concern. Coordinated regional action and targeted interventions, particularly for men and older individuals, are essential to reduce pollution-driven TBLC across Asia.

## 1 Introduction

Tracheal, bronchus, and lung cancer (TBLC) remains a major global health challenge in the 21st century, with an estimated 1.82 million deaths in 2022 (1). Asia, home to 60% of the world’s population (2), faces a particularly concerning epidemiological burden due to its rapid urbanization, industrialization, and significant demographic changes (3, 4). Limited prevention efforts, delayed diagnoses, and unequal treatment access further contribute to Asia’s lower survival rates relative to high-income nations (5, 6).

The pathogenesis of TBLC is driven by the complex interplay of lifestyle factors, genetic susceptibility, and environmental exposures. With 7 million annual deaths linked to indoor and outdoor exposure, air pollution is now recognized as the leading environmental risk factor for global disease burden and ranks second to tobacco in driving non-communicable diseases (7). Ambient particulate matter pollution (APMP), particularly fine particulate matter (PM) with an aerodynamic diameter of less than 2.5 micrometers (PM_2.5_), is a key component of ambient air pollution. It is primarily sourced from vehicle emissions, industrial activities, and power generation. Given its strong association with lung cancer (8), PM_2.5_ has been classified as a Group 1 carcinogen by the International Agency for Research on Cancer (IARC) (9). The World Health Organization (WHO) updated its global air quality guidelines in 2021, with a recommendation that the annual mean PM_2.5_ level should not exceed 5 μg/m^3^ (10). Similarly, household air pollution (HAP) from the burning of solid fuels—such as wood, coal, coal or charcoal—for cooking and heating represents another significant environmental risk factor for TBLC (11). This form of pollution is pervasive in many Asian households, particularly in rural and low-income settings where access to clean energy alternatives remains limited (11, 12).

Numerous previous studies have investigated the global burden of cancer attributable to air pollution, with several highlighting the particular severity in Asia. For example, the Global Burden of Disease Study (GBD) 2019 estimated that ambient PM_2.5_ exposure caused approximately 7.02 million disability-adjusted life years (DALYs) globally in 2019 (13). The burden was notably higher in Asia and Africa compared to the Americas and Europe (13). However, comprehensive studies examining the combined burden of TBLC due to both APMP and HAP across 34 countries and territories in Asia remain scarce. Currently, Asia’s efforts to combat TBLC are closely linked to its air quality crisis. Yet, many countries remain resource-constrained in attaining the United Nations Sustainable Development Goals (SDGs), including SDG 3.4 (reducing premature mortality from non-communicable diseases), SDG 3.9 (lowering pollution-related morbidity and mortality), and SDG 7.1 (ensuring universal access to clean fuels) (14). This gap underscores the need for a study that integrates the latest data to assess the TBLC burden attributable to these environmental risk factors across Asia.

In this study, we conducted a secondary analysis of the most recent 2021 GBD data to quantify the burden of TBLC attributable to APMP and HAP across 34 countries and territories from 1990 to 2021. Stratified analyses were performed to assess variations by age, sex, and location. These findings offer critical insights for developing evidence-based interventions to reduce the TBLC burden caused by air pollution, thereby improving population health, and contributing to the achievement of SDGs.

## 2 Methods

### 2.1 Data sources

This secondary analysis was conducted using freely accessible data from the GBD 2021 results tools (http://ghdx.healthdata.org/gbd-results-tool), which quantifies the burden of 371 diseases and injuries and 88 risk factors in 204 countries and territories from 1990 to 2021 (15, 16). The GBD 2021 is widely recognized as the highest-quality repository of population-health metrics due to its harmonization of over 38,000 distinct data sources, including vital registration systems, cancer registries, verbal autopsy studies, household surveys, and administrative records, through uniform case definitions and a standardized cause hierarchy. Mortality is modeled with the Cause-of-Death Ensemble Model (CODEm), which tests hundreds of candidate models with diverse covariate sets and selects the best-performing ensemble on the basis of out-of-sample predictive validity. Non-fatal outcomes are estimated with DisMod-MR 2.1, a Bayesian meta-regression that enforces internal consistency among incidence, prevalence, remission, and excess mortality. Spatiotemporal Gaussian-process regression borrows strength across neighboring locations and calendar years where primary data are sparse. Rigorous outlier detection, cross-validation, and covariate trimming are implemented at each stage, and final estimates are propagated as 1000 draws to generate 95% uncertainty intervals. Within the GBD 2021 framework, particulate matter pollution (PMP) is classified into two major categories: APMP and HAP. APMP, defined by the population-weighted annual average mass concentration of particles with an aerodynamic diameter less than PM_2.5_ in a cubic meter of air, is derived from an integration of data from satellite aerosol measurements, ground-based monitoring stations, chemical transport models, population distribution data, and land-use patterns (13, 16, 17). HAP is defined as the percentage of the population using solid fuels (coal, crop residues, firewood, cow dung, and charcoal) (13, 16, 18, 19). The calculation is based on data from household surveys (e.g., Demographic and Health Surveys, Multiple Indicator Cluster Surveys), population censuses, WHO’s Household Energy Database, and other relevant sources, including country-specific surveys (16, 18, 19). Additionally, population data used for calculations were obtained from the Gridded Population of the World database, which provides a comprehensive and high-resolution gridded dataset (17). In the GBD comparative risk assessment framework, risks and outcomes are attributed to the same calendar year, with exposure being concurrently associated with outcomes (16). Comprehensive descriptions of these methods are available in the GBD 2021 publication (15, 16, 18, 20, 21). Concurrently, 34 Asian countries and territories are categorized into five GBD-defined regions: High-Income Asia Pacific, East Asia, Southeast Asia, Central Asia, South Asia (Supplementary Table S1) (22). Geospatial data were extended to the continental level, covering Asia, the Americas, Europe, and Africa, to enhance the comprehensiveness of the analysis. TBLC, which is characterized by tumors within the trachea, bronchus, or lung, was classified using specific International Classification of Diseases (ICD) codes. The GBD classification included ICD-10 codes C33, C34-C34.92, and ICD-9 codes 162–162.9, 209.21, V10.1–V10.20, V16.1–V16.2, V16.4–V16.40 (20). DALYs, which quantify the total years of healthy life lost from disease onset to death, were used to define disease burden in this study. This measure includes years of life lost, and years lived with disability. The relevant DALYs numbers and rates were extracted for analysis. Individuals under 25 years of age were excluded from the analysis due to the absence of data on deaths from TBLC attributable to PMP. Subsequently, the remaining age group data were stratified into 5-year intervals ranging 25–29 years to 70–74 years, with an additional group for those aged 75 + years.

### 2.2 Statistical analysis

Age-standardized DALY rates (ASDRs) per 100,000 individuals aged 25 to 75+ years were estimated using the direct standardization method, following Equation 1 (23).


(1) $$\frac{\sum_{i=1}^{N}\alpha_{i}W_{i}}{\sum_{i=1}^{N}W_{i}}$$

In the equation, α_i_ signifies the age-specific rate for the ith age group, while *W_i_* represents the population count for that age group according to the GBD 2021 standard population. *N* indicates the total number of age groups.

Joinpoint regression analysis was employed to examine the temporal trends of ASDRs at the GBD regional, continental, and national levels. It detects points of significant trend changes (i.e., joinpoints), divides the overall trend into multiple subsegments accordingly, and assesses the epidemiological trend of each subsegment using the annual percentage change (APC) and 95% confidence interval (CI) (24). A maximum of 5 joinpoints was permitted based on the number of data points. Statistical significance was evaluated using the Monte Carlo Permutation method. The average annual percentage change (AAPC) was computed as a weighted average of the APCs, with weights corresponding to the length of each joinpoint segment. The assessment of temporal trends followed these criteria: positive values for both the APC/AAPC estimate and the lower 95% confidence boundary indicated an increasing trend, whereas negative values for both the estimate and the upper 95% confidence boundary suggested a decreasing trend. Trends were considered stable in all remaining cases. Decomposition analyses were conducted to investigate the drivers of DALYs changes between 1990 and 2021, evaluating the contributions of population aging, population growth, and epidemiological changes. The approach utilized Das Gupta’s decomposition framework, which required isolating the contribution of each factor while holding the other two constant (25).

Statistical analyses were performed using R (version 4.4.1) and the Joinpoint Regression Program (version 5.2.0.0; National Cancer Institute, Bethesda, MD, United States) (24, 26).

### 2.3 Ethics statement

Ethical approval and consent were not required, as this study utilized publicly available, aggregated, and de-identified secondary data.

## 3 Results

### 3.1 Burden trends at world regional levels

#### 3.1.1 APMP

**Figure 1:**
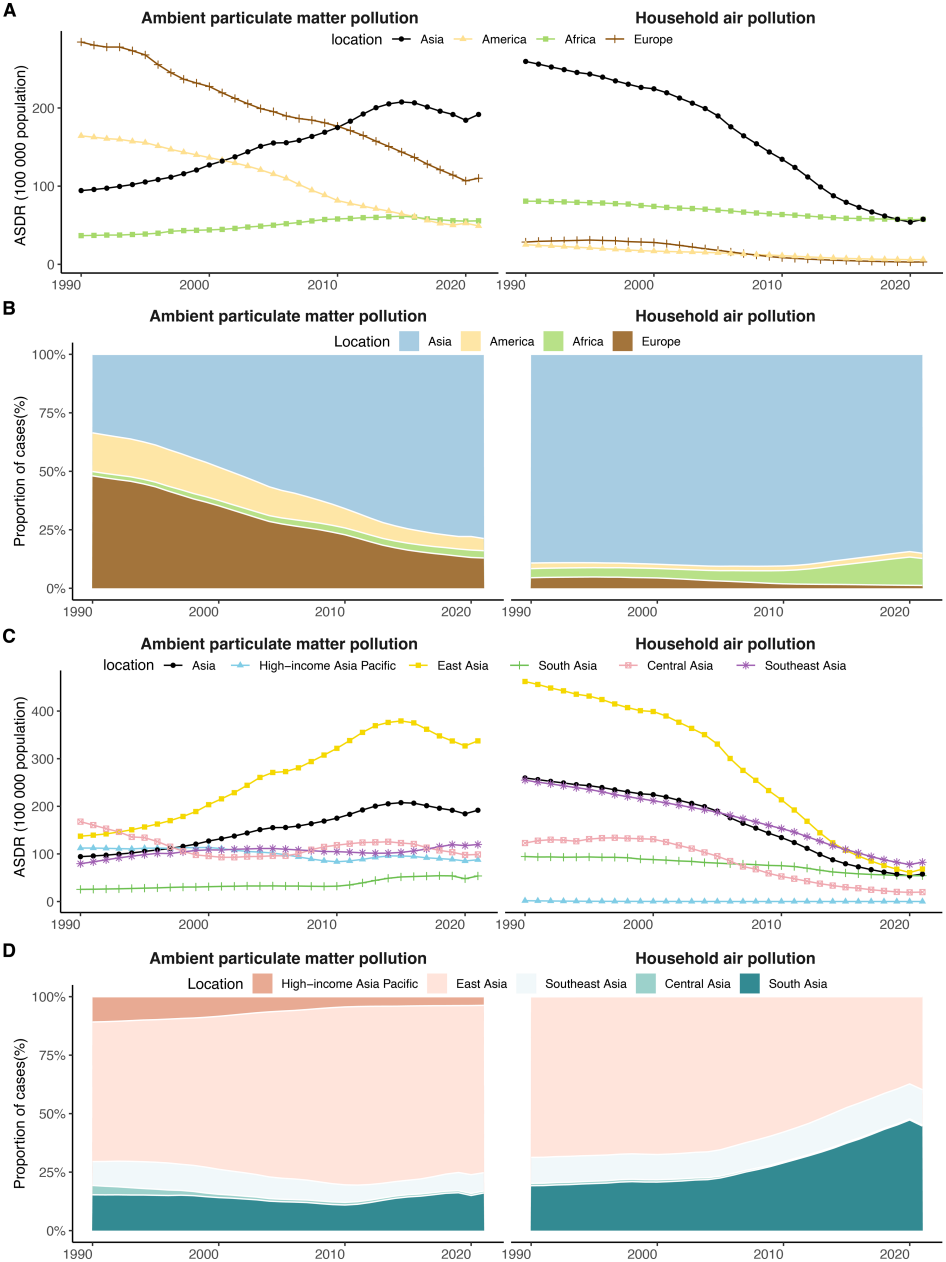
The temporal trends in ASDR and the proportion of DALYs for TBLC attributed to APMP and HAP from 1990 to 2021 across four world regions and five Asia GBD regions. **(A)** The temporal trends in ASDR for TBLC across four world regions. **(B)** The proportion of TBLC-related DALYs across four world regions. **(C)** The temporal trends in ASDR for TBLC across five Asia GBD regions. **(D)** The proportion of TBLC-related DALYs across five Asia GBD regions. DALYs, Disability-Adjusted Life Years; ASDR, Age-Standardized DALY Rate; TBLC, Tracheal, bronchus, and lung cancer; APMP, Ambient Particulate Matter Pollution; HAP, Household Air Pollution.

In 2021, Asia demonstrated the largest DALYs attributable to APMP across four world regions, followed by Europe, America, and Africa (Table 1). From 1990 to 2021, the DALYs in Asia increased from 1,120,408 to 5,477,835 (Table 1 and Supplementary Figure S1), with the proportion rising from 33.56% to 78.73% among the four continents (Figure 1 and Supplementary Table S2). Significantly, the ASDR due to APMP in Asia advanced from third place globally in 1990 to the highest in 2021, with an AAPC of 2.28% (95% CI: 1.96% to 2.61%) (Table 1 and Figure 1). In contrast, the ASDR in America and Europe demonstrated a downward trend, accompanied by AAPCs of −3.77% (95% CI: −4.22% to −3.31%) and −3.06% (95% CI: −3.34% to −2.78%), respectively (Table 1 and Figure 1).

**Table 1:** DALYs and ASDR of TBLC attributed to APMP and HAP in 1990 and 2021 across four world regions and five GBD regions, and the AAPC from 1990 to 2021.

Location	DALYs in 1990 (95%CI)	DALYs in 2021 (95%CI)	ASDR in 1990 (per 100,000 population, 95%CI)	ASDR in 2021 (per 100,000 population, 95%CI)	AAPC of ASDR (%, 95CI)
Ambient particulate matter pollution					
World regional levels					
Asia	1,120,408 (586,864 to 1,862,871)	5,477,835 (3,224,174 to 7,756,353)	94.32 (49.48 to 156.75)	191.70 (112.90 to 271.54)	2.28 (1.96 to 2.61)
America	552,186 (268,394 to 909,444)	363,386 (201,883 to 553,735)	164.37 (79.98 to 270.65)	49.13 (27.35 to 74.78)	−3.77 (−4.22 to −3.31)
Africa	62,095 (37,622 to 90,577)	216,415 (133,986 to 310,873)	36.68 (22.23 to 53.51)	55.61 (34.48 to 79.69)	1.28 (0.91 to 1.66)
Europe	1,603,675 (907,877 to 2,410,170)	900,434 (533,738 to 1,304,860)	284.36 (161.21 to 427.36)	110.12 (65.35 to 159.60)	−3.06 (−3.34 to −2.78)
GBD regional levels					
High-income Asia Pacific	127,283 (32,914 to 259,109)	216,452 (110,965 to 341,262)	112.25 (28.91 to 228.77)	87.75 (45.40 to 137.63)	−0.82 (−1.07 to −0.57)
East Asia	701,440 (307,184 to 1,309,526)	4,168,708 (2,402,587 to 5,991,763)	137.25 (60.06 to 256.25)	337.47 (194.67 to 485.40)	2.92 (2.45 to 3.39)
South Asia	180,009 (89,173 to 299,528)	936,965 (537,127 to 1,425,131)	25.52 (12.60 to 42.57)	53.69 (30.75 to 81.67)	2.34 (1.86 to 2.81)
Central Asia	46,880 (17,758 to 88,269)	48,691 (29,289 to 71,055)	167.80 (63.58 to 316.07)	98.80 (59.44 to 144.17)	−1.77 (−2.34 to −1.20)
Southeast Asia	119,962 (51,539 to 220,585)	456,382 (248,343 to 695,227)	79.49 (34.17 to 146.31)	119.78 (65.39 to 182.12)	1.31 (1.12 to 1.49)
Household air pollution					
World regional levels					
Asia	3,135,620 (2,021,418 to 4,320,800)	1,670,460 (585,114 to 4,116,702)	259.51 (167.31 to 357.37)	57.70 (20.11 to 142.68)	−4.82 (−5.07 to −4.56)
America	84,590 (33,298 to 175,048)	44,076 (15,076 to 117,852)	25.16 (9.89 to 52.10)	6.09 (2.09 to 16.21)	−4.54 (−4.86 to −4.21)
Africa	136,573 (86,771 to 198,110)	226,386 (137,616 to 333,034)	80.78 (51.36 to 117.11)	57.53 (35.11 to 84.16)	−1.12 (−1.19 to −1.05)
Europe	158,357 (35,779 to 506,100)	23,949 (1,497 to 147,674)	28.44 (6.42 to 90.77)	2.99 (0.19 to 18.33)	−7.08 (−7.39 to −6.77)
GBD regional levels					
High-income Asia Pacific	1,957 (135 to 11,874)	99 (0 to 699)	1.72 (0.12 to 10.43)	0.04 (0.00 to 0.27)	−11.64 (−12.12 to −11.16)
East Asia	2,375,963 (1,516,001 to 3,307,802)	84,3081 (164,667 to 2,683,968)	462.12 (295.04 to 642.57)	68.20 (13.30 to 217.12)	−6.09 (−6.51 to −5.68)
South Asia	664,672 (424,478 to 905,703)	948,315 (498,783 to 1,575,684)	94.52 (60.32 to 128.90)	54.29 (28.51 to 90.26)	−1.77 (−2.03 to −1.51)
Central Asia	34,051 (12,492 to 75,777)	9,852 (3,393 to 25,162)	122.84 (45.20 to 272.68)	19.86 (6.79 to 51.02)	−5.84 (−6.30 to −5.38)
Southeast Asia	384,077 (236,790 to 542,299)	319,631 (120,420 to 632,493)	254.93 (157.06 to 359.44)	82.41 (31.07 to 163.42)	−3.64 (−3.72 to −3.56)

DALYs, Disability-Adjusted Life Years; ASDR, Age-standardized DALYs rate; TBLC, Tracheal, bronchus, and lung cancer; APMP, Ambient Particulate Matter Pollution; HAP, Household Air Pollution; AAPC, average annual percentage change; CI, confidence interval.

#### 3.1.2 HAP

Among four world regions in 2021, Asia recorded the largest DALYs attributable to HAP, with Africa, America, and Europe ranking subsequently (Table 1). Between 1990 and 2021, Asia experienced a decline from 3,135,620 to 1,670,460 (Table 1 and Supplementary Figure S1), and its share among the four continents fell from 89.20% to 85.02% (Figure 1 and Supplementary Table S2). The ASDR for HAP decreased across all world regions during the study period, with the most pronounced reduction noted in Europe (AAPC = −7.08%, 95% CI: −7.39% to −6.77%) (Table 1 and Figure 1). Initially, in 1990, the ASDR for HAP in Asia was significantly higher than that of the other three world regions. However, by 2021, this rate had decreased to a level comparable to that of Africa (Table 1 and Figure 1).

### 3.2 Burden trends at GBD regional leaves

#### 3.2.1 APMP

Among five GBD regions, East Asia exhibited the highest DALYs attributable to APMP in 2021, followed by South Asia and Southeast Asia (Table 1). Between 1990 and 2021, the proportion of DALYs attributed to APMP in East Asia increased significantly from 59.67% to 71.94% (Figure 1 and Supplementary Table S3). During this period, the ASDR for APMP-related TBLC demonstrated an upward trend in East Asia, South Asia, and Southeast Asia, while High-income Asia Pacific and Central Asia experienced a decline (Table 1 and Figure 1). By 2021, East Asia reported the highest ASDR, in contrast to South Asia, which recorded the lowest rate (Table 1 and Figure 1).

#### 3.2.2 HAP

In 2021, South Asia and East Asia represented the largest HAP-related DALYs among the GBD regions, contributing 44.71% and 39.75%, respectively (Figure 1 and Supplementary Table S3). From 1990 to 2021, all GBD regions experienced a decline in HAP-related ASDR, with the most substantial decrease in the High-income Asia Pacific (AAPC = −11.64%, 95% CI: −12.12% to −11.16%) and the least in South Asia (AAPC = −1.77%, 95% CI: −2.03% to −1.51%) (Table 1 and Figure 1). By 2021, Southeast Asia recorded the highest rate, followed by East Asia and South Asia (Table 1 and Figure 1).

### 3.3 Burden trends at national levels

#### 3.3.1 APMP

**Figure 2:**
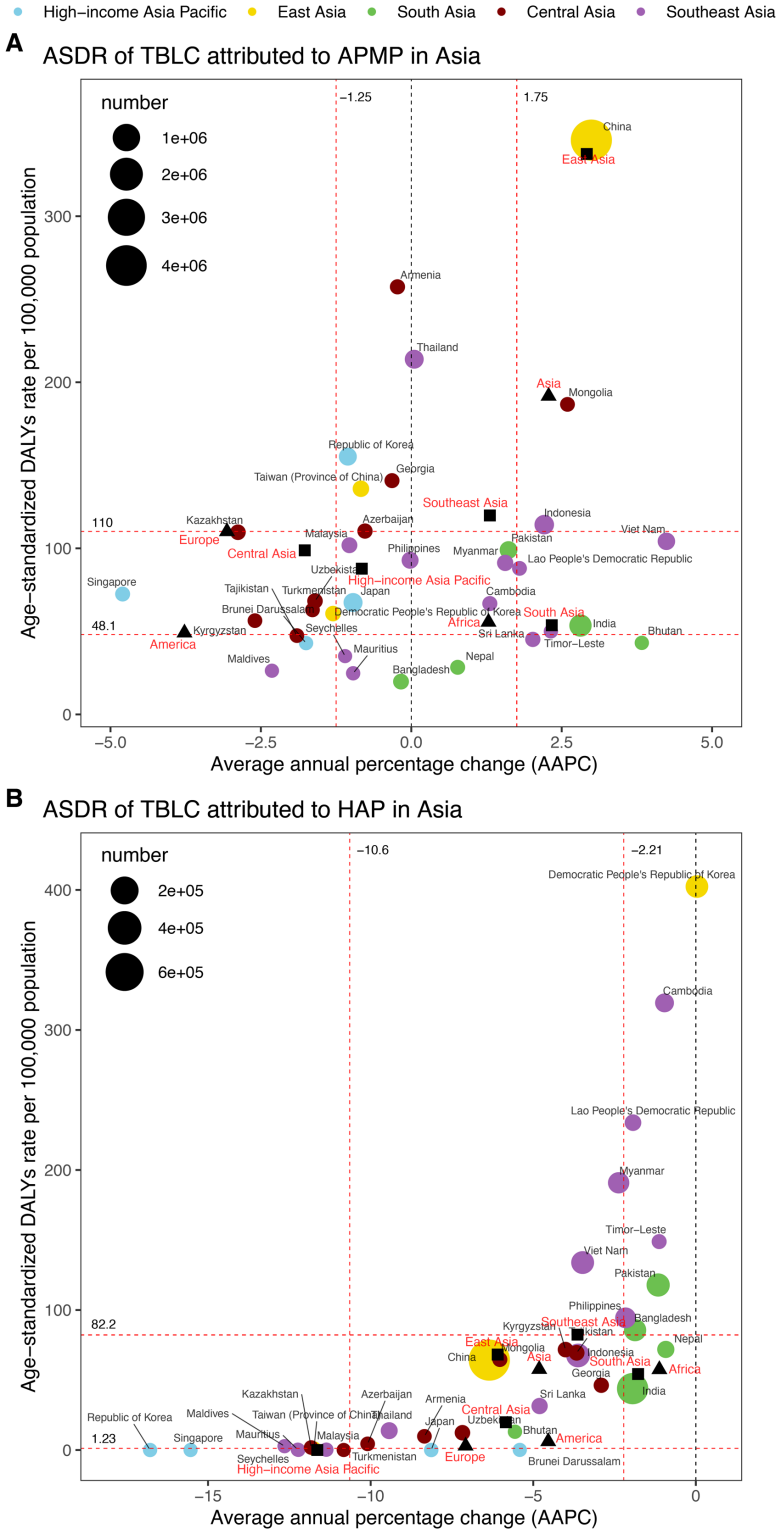
ASDR and DALYs of TBLC attributed to APMP and HAP in 2021, and the AAPC of ASDR from 1990 to 2021 across four world regions and five Asia GBD regions. **(A)** ASDR and DALYs of TBLC attributed to APMP. **(B)** ASDR and DALYs of TBLC attributed to HAP. The circles’ colors identify the GBD region of each country or territory, while their sizes reflect DALYs values. Red dashed lines denote the tertiles of AAPC or ASDR. Solid black squares denote the five Asia GBD regions, while black-filled triangles represent four global regions. ASDR, Age-standardized DALYs rate; DALYs, Disability-Adjusted Life Years; TBLC, Tracheal, bronchus, and lung cancer; APMP, Ambient Particulate Matter Pollution; HAP, Household Air Pollution; AAPC, Average annual percentage change.

In 2021, China bore the greatest burden of APMP-attributable DALYs (4,125,753, 95% CI: 2,374,169 to 5,934,144), followed by India (375,887, 95% CI: 219,776 to 562,794) and Indonesia (163,693, 95% CI: 76,558 to 272,433) (Figure 2 and Supplementary Table S4). China also exhibited the highest ASDR from APMP at 345.81(95% CI: 199.13 to 497.72) per 100,000 population, with Thailand (213.87, 95% CI: 120.84 to 334.21 per 100,000 population) and Mongolia (186.68, 95% CI: 81.62 to 299.29 per 100,000 population) ranking subsequently (Figure 2; Supplementary Table S4; Supplementary Figure S2). From 1990 to 2021, 13 countries experienced rising APMP-related ASDRs, with Viet Nam recording the most rapid (AAPC = 4.24%, 95% CI: 4.05% to 4.43%). In contrast, 16 countries observed declines, with Singapore documenting the most significant reduction (AAPC = −4.80%, 95% CI: −5.82% to −3.76%) (Figure 2; Supplementary Table S4; Supplementary Figure S3).

#### 3.3.2 HAP

In 2021, China recorded the highest HAP-related DALYs (766,682, 95% CI: 106,434 to 2,596,250), followed by India (309,380, 95% CI: 156,704 to 534,949) and Indonesia (96,125, 95% CI: 28,632 to 223,452) (Figure 2 and Supplementary Table S4). The Democratic People’s Republic of Korea reported the highest ASDR (402.40, 95% CI: 212.35 to 675.63 per 100,000 population), with Cambodia (319.33, 95% CI: 172.17 to 506.60 per 100,000 population) and Lao People’s Democratic Republic (233.79, 95% CI: 92.62 to 428.03 per 100,000 population) ranking next (Figure 2; Supplementary Table S4; Supplementary Figure S4). Between 1990 and 2021, ASDR declined in 33 countries, with the Republic of Korea experiencing the sharpest decrease (AAPC = −16.78%, 95% CI: −17.09% to −16.47%). The Democratic People’s Republic of Korea remained stable (AAPC = 0.03%, 95% CI: −0.02% to 0.09%), and no country showed an upward trend (Figure 2; Supplementary Table S4; Supplementary Figure S5).

### 3.4 Decomposition analysis of TBLC DALYs

**Figure 3:**
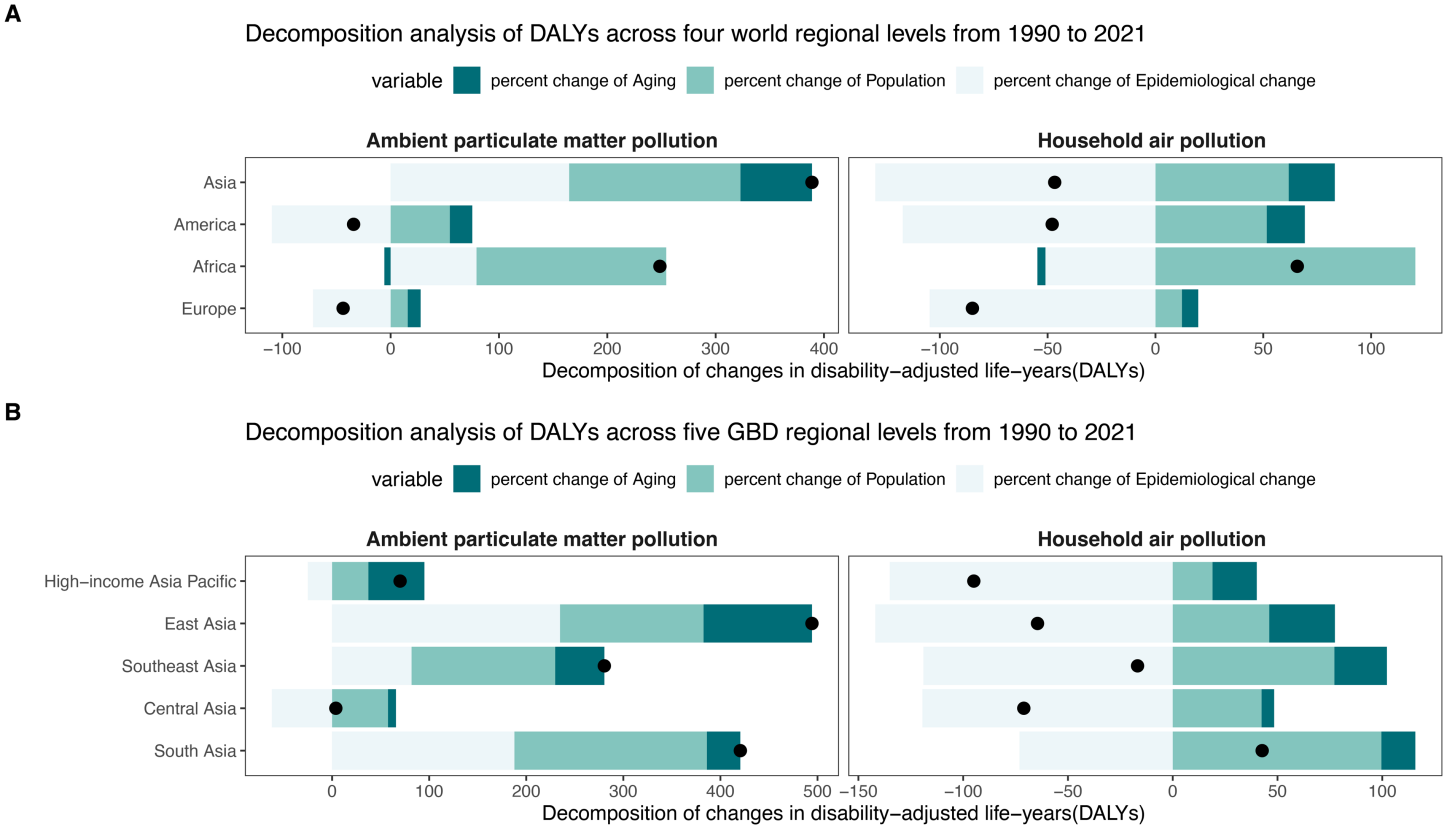
The proportions of changed DALYs for TBLC according to population aging, population growth, and epidemiological change between 1990 and 2021 across four world regions **(A)** and five Asia GBD regions **(B)**. DALY, Disability-Adjusted Life Years; TBLC, Tracheal, bronchus, and lung cancer; APMP, Ambient Particulate Matter Pollution; HAP, Household Air Pollution. The black dots in the figure denote the total DALYs change between 1990 and 2021.

The findings from the decomposition analysis revealed that population growth and epidemiologic change were the primary contributors to DALYs attributable to APMP in Asia (Figure 3). However, epidemiologic change had pronounced effects in the Americas (−109.55%), Europe (−71.56%), and East Asia (234.75%) (Supplementary Table S5). For HAP, epidemiologic change was the predominant factor, exerting a negative effect across all regions, with the most significant impact recorded in Asia (−129.90%), particularly East Asia (−141.92%) (Supplementary Table S6). In contrast, population growth significantly influenced DALY variations in Africa (120.53%) and South Asia (99.57%). Overall, APMP-related DALYs shown an increasing trend in most regions, whereas a decreasing trend observed in the Americas (−34.19%) and Europe (−43.85%), driven by the stronger absolute negative effect of epidemiologic change. Conversely, HAP-related DALYs generally exhibited a downward trend, though an upward trend was observed in Africa (65.76%) and South Asia (42.67%) due to the greater absolute positive effect of population growth.

### 3.5 Burden trends at different sex groups

**Figure 4:**
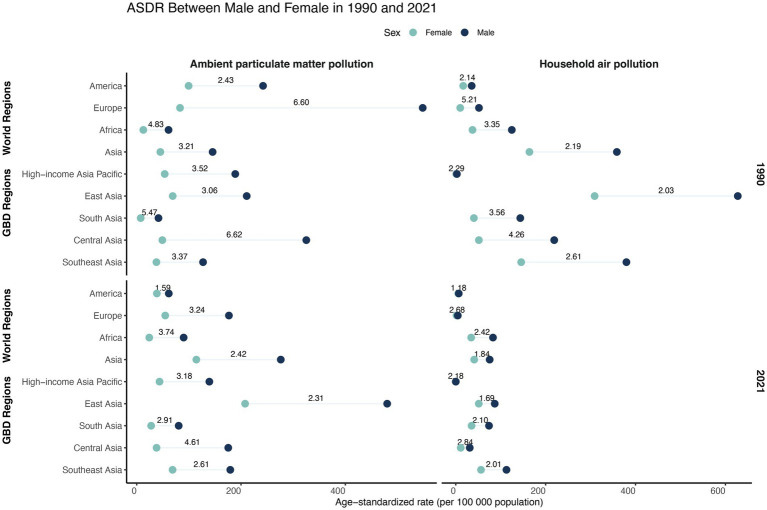
ASDR of TBLC attributed to APMP and HAP by sex in 1990 and 2021, across four world regions and five Asia GBD regions. The numbers on the horizontal line indicate the ratio of male to female ASDR. ASDR, Age-standardized DALYs rate; DALYs, Disability-Adjusted Life Years; TBLC, Tracheal, bronchus, and lung cancer; APMP, Ambient Particulate Matter Pollution; HAP, Household Air Pollution.

Notable sex-based disparities in air pollution-related ASDR were observed in both 1990 and 2021 (Figure 4 and Supplementary Table S7). Males showed higher ASDRs compared to females. The male-to-female ASDR ratio for APMP was generally higher than that for HAP (Figure 4). Additionally, the gender gap in ASDR exhibited a modest reduction over the period from 1990 to 2021 (Figure 4 and Supplementary Table S7).

### 3.6 Burden trends at different age groups

**Figure 5:**
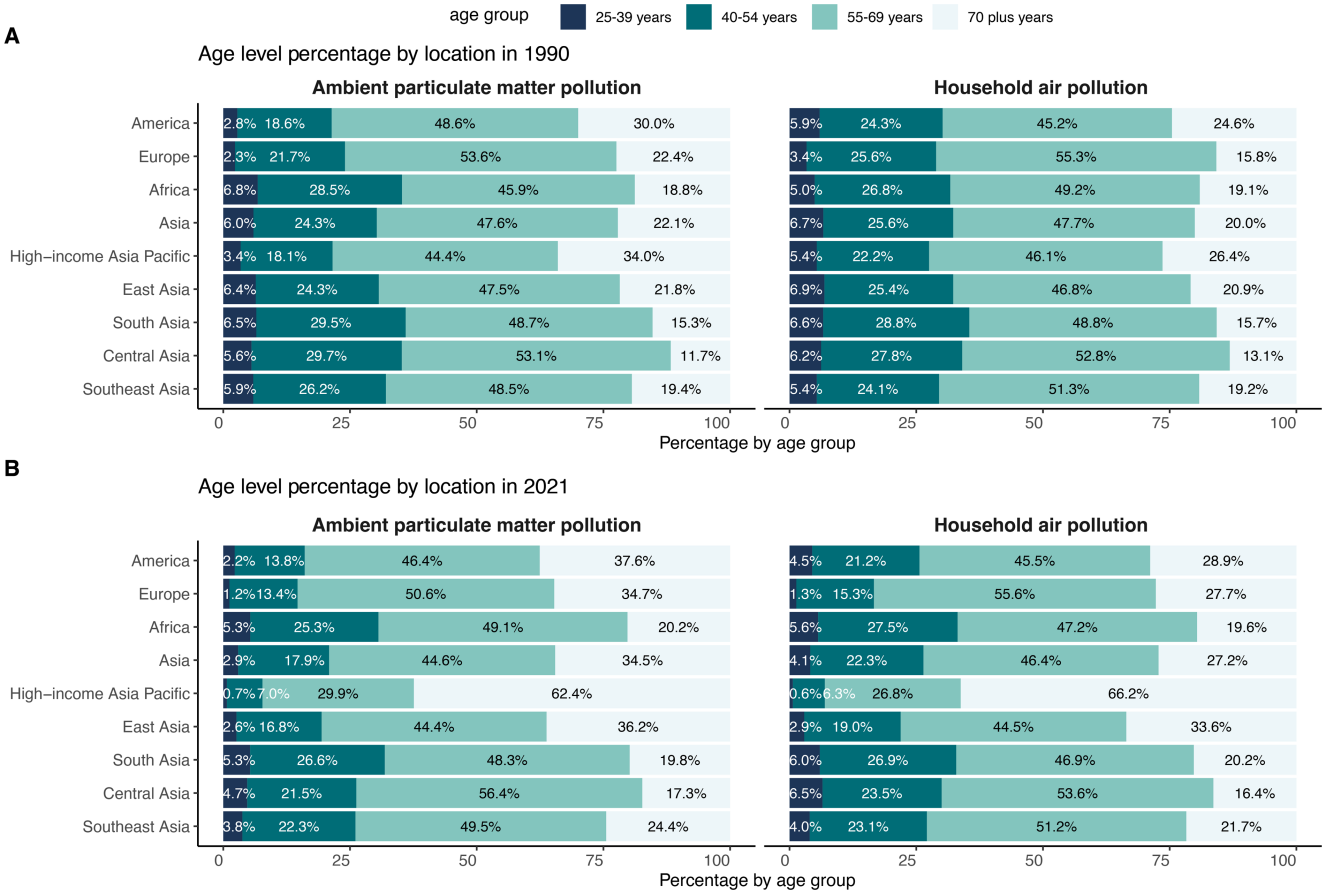
Age group distribution of DALYs across four world regions and five Asia GBD regions in 1990 **(A)** and 2021 **(B)**. DALY, Disability-Adjusted Life Years.

Across all regions, individuals aged 55–69 accounted for nearly half of DALYs in both 1990 and 2021 (Figure 5 and Supplementary Table S8). In High-income Asia Pacific in 2021, however, individuals aged 70 and above accounted for 62.40% and 66.20% of DALYs due to APMP and HAP, respectively.

**Figure 6:**
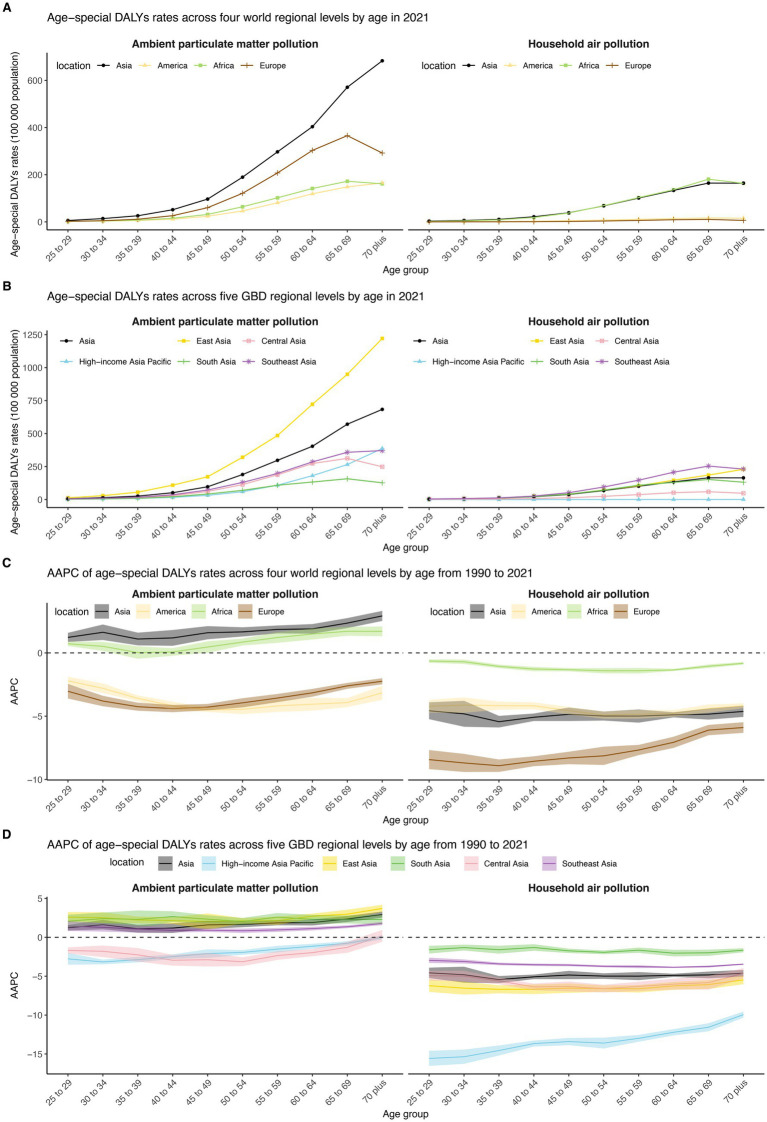
DALY rates of TBLC attributed to APMP and HAP in different age groups in 2021, and their AAPC from 1990 to 2021, across four world regions and five Asia GBD regions. **(A)** DALY rates of TBLC in different age groups in 2021 across four world regions. **(B)** DALY rates of TBLC in different age groups in 2021 across five Asia GBD regions. **(C)** AAPC of DALY rates in different age groups from 1990 to 2021 across four world regions. **(D)** AAPC of DALY rates in different age groups from 1990 to 2021 across five Asia GBD regions. DALY, Disability-Adjusted Life Years; TBLC, Tracheal, bronchus, and lung cancer; APMP, Ambient Particulate Matter Pollution; HAP, Household Air Pollution.

Across the four world regions, crude DALY rates for both APMP and HAP rose steadily with age (Figure 6 and Supplementary Table S9). In 2021, Asia recorded the highest APMP burden, while Asia and Africa shared the highest HAP burden in every age group. Between 1990 and 2021, HAP-related DALY rates fell in all world regions—most sharply in Europe—whereas APMP-related rates declined in the Americas and Europe but increased across nearly every age group in Asia and Africa. Within the five Asia-specific GBD regions, East Asia posted the highest crude DALY rate for APMP in 2021, whereas Southeast Asia led for HAP (Figure 6 and Supplementary Table S9). From 1990 to 2021, HAP-related DALY rates dropped most markedly in the High-income Asia Pacific. For APMP, crude DALY rates fell in the High-income Asia Pacific and Central Asia but rose across all age groups in East, South, and Southeast Asia.

## 4 Discussion

The study reveals several key observations regarding the ASDR for TBLC attributed to APMP and HAP from 1990 to 2021, with stratifications by sex, age, and location. As illustrated in Table 1, the ASDR due to APMP has declined significantly in Europe and America, while in Asia, it has risen markedly. The ASDR due to HAP has decreased substantially, falling below that of APMP and reaching levels comparable to those in Africa. In Europe and America, the HAP-related ASDR has remained consistently low. Additionally, DALYs in Asia, particularly those related to APMP, have increased over the past 31 years, indicating a growing health burden. Within Asian subregions in 2021, East Asia recorded the greatest DALYs attributable to APMP, while South Asia and East Asia exhibited the highest HAP-related TBLC burden. Among the 34 Asian countries and territories examined, China reported the highest DALYs for both APMP and HAP exposures. Decomposition analysis showed that the increase in APMP-related DALYs was predominantly driven by population growth and epidemiological changes. Additionally, epidemiological changes were identified as the main drivers of the decline in HAP-related DALYs. Furthermore, ASDR were consistently higher among males, with a more pronounced sex disparities observed in ASDR attributable to APMP. Lastly, middle-aged, and older adult populations emerged as the most vulnerable demographic groups.

Research suggests that PM, particularly PM_2.5_, is a significant risk factor for lung cancer, with cohort studies showing a positive association between long-term PM exposure and TBLC mortality (27–29). In high-income regions such as Europe and America, the APMP-related ASDR for TBLC has declined markedly over recent decades. Similar to the study by Deng et al. (3), the United States and the European Union exhibited a continuous decline in lung cancer-related ASDR due to total PM_2.5_ exposure from 1990 to 2021, with estimated annual percentage change values of −6.01 and −2.91, respectively. This decrease is likely reflective of stringent air quality regulations, technological improvements, and robust public health interventions that have collectively reduced ambient levels of harmful PM (30, 31). Using the GEOS-Chem adjoint model, Gu et al. (32) introduced a novel method to trace the origins of PM_2.5_-linked premature deaths in Europe. Their findings demonstrated that emission mitigation efforts during the first decade of PM_2.5_ regulation (2005–2015) substantially decreased PM_2.5_-related mortality, preventing an estimated 63,538 premature deaths (95% CI: 46,092 to 91,082) across most European countries. The implementation of policies such as the Clean Air Act in the United States and European Union directives has led to decreased emissions from industrial and transportation sources, contributing to lower exposure levels. In contrast, Asia has experienced a significant increase in APMP-related ASDR, with China shouldering the highest burden in East Asia. This upward trend in APMP-related ASDR of TBLC in Asia echoes findings from several epidemiological studies (3, 33). For instance, a spatiotemporal study from Nepal revealed that in the Kathmandu Valley, the average standardized incidence ratio (SIR) of lung cancer associated with PM_2.5_ increased from a minimum of 4.96 in 2012 to a maximum of 7.81 in 2021 (29). Furthermore, a cohort study conducted in China has demonstrated a significant association between high concentrations of PM_2.5_ exposure and an increased incidence and mortality rate of lung cancer (34). Vietnam’s rapid industrial development, spurred by economic growth and foreign investment, may contribute to increased APMP, potentially affecting TBLC rates (35, 36). However, specific data on air quality trends and their impact on health outcomes are necessary to confirm this. These trends reflect both the demographic transitions, including population aging and growth, and the epidemiological shifts influenced by industrial development and urbanization. Interestingly, the implementation of the Action Plan for Air Pollution Prevention and Control (2013–2017) in China resulted in a significant improvement in air quality (37, 38). The national population-weighted annual mean PM_2.5_ concentration decreased from 67.4 μg/m^3^ in 2013 to 45.5 μg/m^3^ in 2017, corresponding to a 32% reduction (38). The lag effect may be at play here, as lung cancer development generally occurs over decades following exposure, meaning that current cases may reflect prior high pollution levels.

In Asia, the ASDR for TBLC due to HAP has decreased substantially, now falling below that of APMP and reaching levels comparable to those in Africa. Decomposition analysis indicates the decline in HAP-related DALYs was primarily driven by epidemiological changes, including expanded access to cleaner energy sources (e.g., liquefied petroleum gas and electricity), and improved household ventilation (39). In high-income regions (Europe and America), HAP-associated ASDR have remained persistently low, consistent with near-universal adoption of clean cooking/heating fuels (solid fuel use <5%) (40). As depicted in Table 1, the TBLC burden linked to HAP in Asia has decreased, but notable regional differences persist. The highest ASDRs attributable to HAP in 2021 were recorded in the Democratic People’s Republic of Korea, Cambodia, and the Lao People’s Democratic Republic. These countries, characterized by lower Socio-Demographic Index levels, continue to rely heavily on biomass fuels for cooking and heating, perpetuating exposure to carcinogenic pollutants. By contrast, high-income Asian countries such as South Korea, Japan, and Singapore have maintained consistently low ASDRs from HAP, with significant declines in South Korea and Singapore. These patterns may be explained by the advanced infrastructure in these countries, which enhances clean energy adoption, as well as the implementation of effective cancer control programs and advanced diagnostic and therapeutic strategies.

Our analysis revealed persistent sex-based disparities, with males exhibiting higher ASDRs for TBLC associated with both APMP and HAP compared to females in both 1990 and 2021. The male-to-female ASDR ratio was more pronounced for APMP than HAP, suggesting differential exposure pathways or biological susceptibilities. Males, in many settings, have greater exposure to outdoor air pollution, potentially due to occupational factors or increased time spent in polluted environments such as commuting or working in industrial areas. Interestingly, despite WHO reports noting that women traditionally have higher exposure to solid fuel smoke from cooking (19), males still exhibited higher ASDRs due to HAP. Anatomical and hormonal differences, including lung capacity and estrogen-mediated protective effects, may contribute to the lower susceptibility of women to air pollution-induced respiratory pathologies (41). The modest reduction in the gender gap from 1990 to 2021 could reflect changing societal roles, with more women entering the workforce and thus facing similar APMP exposure, or improvements in household ventilation reducing men’s HAP exposure. Nonetheless, it highlights the persistence of sex-based disparities that necessitate targeted policy interventions.

Across most Asia regions, individuals aged 55–69 years accounted for nearly half of TBLC DALYs attributable to APMP and HAP, reflecting the cumulative nature of air pollution exposure and the latency period of carcinogenesis. In High-income Asia Pacific, however, adults aged ≥70 years represented over 60% of DALYs in 2021. This observation may be attributable to demographic changes, including population aging and increased life expectancy. This could also indicate past high exposure levels, as older adults were exposed to worse air quality earlier in life, consistent with historical air pollution trends in high-income regions.

### 4.1 Limitation

Several limitations of our study must be acknowledged. First, our research is a secondary analysis based on the GBD database, and the accuracy of our results depends on the quality of the GBD data. In low- and middle-income countries with inadequate vital registration systems, GBD estimates rely on statistical modeling rather than direct measurements. Misdiagnosis or misclassification may introduce bias, thereby underestimating the true TBLC burden. Second, the GBD 2021 study is limited to a specific subset of particulate pollutants, while neglecting other significant outdoor pollutants (e.g., sulfur dioxide, ozone) and indoor combustion-related pollutants (e.g., kerosene smoke, carbon monoxide). These pollutants may independently or synergistically contribute to TBLC risk. Furthermore, GBD 2021 lacks detailed classification of particulate matter, including PM_0.1_, PM_2.5_, PM_10_. At sites where only PM_10_ measurements were available, PM_2.5_ concentrations were estimated using a hierarchy of conversion factors (PM_2.5_/PM_10_ ratios) (17). This is significant because particle size is a key factor influencing deposition in the airways and carcinogenic potential. For instance, PM_2.5_ can bypass mucociliary clearance to reach the alveolar epithelium, generating reactive oxygen species and directly transporting adsorbed polycyclic aromatic hydrocarbons to bronchial cells. In contrast, larger PM_10_ particles predominantly remain in the upper respiratory tract and are less associated with lung cancer (8). Third, the GBD 2021 public version only provides cross-sectional estimates for the same year. The lack of longitudinal exposure data prohibits the application of distributed lag non-linear models, which are necessary to evaluate the potential lagged effects of PM exposure on TBLC burden. Consequently, this may either underestimate or overestimate the mid- to long-term impacts of PM on TBLC (16, 42, 43). Lastly, our study included 34 countries and territories from Central, East, South, and Southeast Asia, as well as high-income Asia-Pacific nations, based on GBD classification. Western Asia is not analyzed because GBD classifies these countries in a separate super-region. However, Western Asia shares notable environmental and epidemiological characteristics with neighboring regions included in our analysis, such as South Asia. Excluding these countries could result in gaps in understanding the *trans*-regional patterns of TBLC burden associated with PMP. Nevertheless, our findings deliver a rigorous, data-driven evaluation of APM- and HAP-induced TBLC burden across major Asian populations, serving as a foundational reference for evidence-based policymaking in environmental health.

## 5 Conclusion

In conclusion, our findings highlight an urgent public health challenge in Asia: although improvements in HAP have reduced the burden of lung cancer, APMP continues to pose a significant threat. Without stricter air quality regulations and cleaner energy transitions, ongoing urbanization, and industrial growth, coupled with population growth, may increase TBLC burdens attributable to air pollution. Strengthening international cooperation and focusing on high-risk populations, particularly men, middle-aged and older adults, and low-income groups, are critical strategies to reduce the future burden of TBLC.

## Data Availability

The data employed in this research are accessible to the public at: http://ghdx.healthdata.org/gbd-results-tool. Registration is required to access and download the datasets.

## References

[1] Bray F Laversanne M Sung H Ferlay J Siegel RL Soerjomataram I. Global cancer statistics 2022: GLOBOCAN estimates of incidence and mortality worldwide for 36 cancers in 185 countries. CA Cancer J Clin. (2024) 74:229–63. doi: 10.3322/caac.21834, PMID: 38572751

[2] Dalakoti M Lin NHY Yap J Cader A Dipanker P Lee D. Primary prevention of cardiovascular disease in Asia: challenges: a narrative review. JACC Adv. (2025) 4:101670. doi: 10.1016/j.jacadv.2025.101670, PMID: 40117691 PMC11976236

[3] Deng Y Li Z Zhang P Yang Y Xie Y Cheng Y. Global, regional and national burden of lung cancer attributable to PM(2.5) air pollution: trends from 1990 to 2021 with projections to 2045. J Environ Manag. (2025) 390:126216. doi: 10.1016/j.jenvman.2025.126216, PMID: 40561923

[4] Ding J Guo W Xue Q Cheng G Zhang L Wu D. Global and East Asia tracheal, bronchus, and lung cancer trend analysis from 1990 to 2021 and forecast trend from 2021 to 2035. Front Oncol. (2025) 15:1542067. doi: 10.3389/fonc.2025.1542067, PMID: 40171264 PMC11960504

[5] Lam DC Liam CK Andarini S Park S Tan DSW Singh N. Lung cancer screening in Asia: an expert consensus report. J Thorac Oncol. (2023) 18:1303–22. doi: 10.1016/j.jtho.2023.06.014, PMID: 37390982

[6] Chiu CH Yang PC. Challenges of lung cancer control in Asia. EClinicalMedicine. (2024) 74:102706. doi: 10.1016/j.eclinm.2024.102706, PMID: 39764183 PMC11701479

[7] Campbell-Lendrum D Prüss-Ustün A. Climate change, air pollution and non-communicable diseases. Bull World Health Organ. (2019) 97:160–1. doi: 10.2471/blt.18.224295, PMID: 30728622 PMC6357572

[8] Neupane BK Acharya BK Cao C Xu M Bhattarai H Yang Y. A systematic review of spatial and temporal epidemiological approaches, focus on lung cancer risk associated with particulate matter. BMC Public Health. (2024) 24:2945. doi: 10.1186/s12889-024-20431-x, PMID: 39448953 PMC11515550

[9] Hamra GB Guha N Cohen A Laden F Raaschou-Nielsen O Samet JM. Outdoor particulate matter exposure and lung cancer: a systematic review and meta-analysis. Environ Health Perspect. (2014) 122:906–11. doi: 10.1289/ehp/1408092, PMID: 24911630 PMC4154221

[10] Pérez Velasco R Jarosińska D. Update of the WHO global air quality guidelines: systematic reviews – an introduction. Environ Int. (2022) 170:107556. doi: 10.1016/j.envint.2022.107556, PMID: 36395555 PMC9720155

[11] Gordon SB Bruce NG Grigg J Hibberd PL Kurmi OP Lam KB. Respiratory risks from household air pollution in low and middle income countries. Lancet Respir Med. (2014) 2:823–60. doi: 10.1016/s2213-2600(14)70168-7, PMID: 25193349 PMC5068561

[12] Chafe ZA Brauer M Klimont Z Van Dingenen R Mehta S Rao S. Household cooking with solid fuels contributes to ambient PM2.5 air pollution and the burden of disease. Environ Health Perspect. (2014) 122:1314–20. doi: 10.1289/ehp.1206340, PMID: 25192243 PMC4256045

[13] Chen J Cui Y Deng Y Xiang Y Chen J Wang Y. Global, regional, and national burden of cancers attributable to particulate matter pollution from 1990 to 2019 and projection to 2050: worsening or improving? J Hazard Mater. (2024) 477:135319. doi: 10.1016/j.jhazmat.2024.135319, PMID: 39059291

[14] United Nations. Sustainable Development Goals (2016). Available online at: https://www.un.org/sustainabledevelopment/ (accessed January 1, 2025)

[15] GBD 2021 Diseases and Injuries Collaborators. Global incidence, prevalence, years lived with disability (YLDs), disability-adjusted life-years (DALYs), and healthy life expectancy (HALE) for 371 diseases and injuries in 204 countries and territories and 811 subnational locations, 1990-2021: a systematic analysis for the global burden of disease study 2021. Lancet. (2024) 403:2133–61. doi: 10.1016/s0140-6736(24)00757-8 38642570 PMC11122111

[16] GBD 2021 Risk Factors Collaborators. Global burden and strength of evidence for 88 risk factors in 204 countries and 811 subnational locations, 1990-2021: a systematic analysis for the global burden of disease study 2021. Lancet. (2024) 403:2162–203. doi: 10.1016/s0140-6736(24)00933-4 38762324 PMC11120204

[17] Institute for Health Metrics and Evaluation. Ambient particulate matter pollution (2024). Available online at: https://www.healthdata.org/gbd/methods-appendices-2021/ambient-particulate-matter-pollution (accessed January 15, 2025)

[18] GBD 2021 HAP Collaborators. Global, regional, and national burden of household air pollution, 1990-2021: a systematic analysis for the global burden of disease study 2021. Lancet. (2025) 405:1167–81. doi: 10.1016/s0140-6736(24)02840-x 40118081 PMC11971481

[19] Institute for Health Metrics and Evaluation. Household air pollution(2024). Available online at: https://www.healthdata.org/gbd/methods-appendices-2021/household-air-pollution (accessed January 15, 2025)

[20] GBD 2021 Causes of Death Collaborators. Global burden of 288 causes of death and life expectancy decomposition in 204 countries and territories and 811 subnational locations, 1990-2021: a systematic analysis for the global burden of disease study 2021. Lancet. (2024) 403:2100–32. doi: 10.1016/s0140-6736(24)00367-2 38582094 PMC11126520

[21] GBD 2021 Diarrhoeal Diseases Collaborators. Global age-sex-specific mortality, life expectancy, and population estimates in 204 countries and territories and 811 subnational locations, 1950-2021, and the impact of the COVID-19 pandemic: a comprehensive demographic analysis for the global burden of disease study 2021. Lancet. (2024) 403:1989–2056. doi: 10.1016/s0140-6736(24)00476-8 38484753 PMC11126395

[22] Murray CJ Ezzati M Flaxman AD Lim S Lozano R Michaud C. GBD 2010: design, definitions, and metrics. Lancet. (2012) 380:2063–6. doi: 10.1016/s0140-6736(12)61899-6, PMID: 23245602

[23] GBD 2019 Demographics Collaborators. Global age-sex-specific fertility, mortality, healthy life expectancy (HALE), and population estimates in 204 countries and territories, 1950-2019: a comprehensive demographic analysis for the global burden of disease study 2019. Lancet. (2020) 396:1160–203. doi: 10.1016/s0140-6736(20)30977-6 33069325 PMC7566045

[24] Kim HJ Fay MP Feuer EJ Midthune DN. Permutation tests for joinpoint regression with applications to cancer rates. Stat Med. (2000) 19:335–51. doi: 10.1002/(sici)1097-0258(20000215)19:3<335::aid-sim336>3.0.co;2-z, PMID: 10649300

[25] Das Gupta P. Standardization and decomposition of rates: a user’s manual. Washington (D.C.): US Department of Commerce (1993).

[26] National Cancer Institute. Joinpoint Trend Analysis Software (2024). Available online at: https://surveillance.cancer.gov/joinpoint/ (accessed June 9, 2024)

[27] Vanoli J de la Cruz Libardi A Sera F Stafoggia M Masselot P Mistry MN. Long-term associations between time-varying exposure to ambient PM 2.5 and mortality: an analysis of the UK biobank. Epidemiology. (2025) 36:1–10. doi: 10.1097/ede.0000000000001796, PMID: 39435892 PMC13016441

[28] Chen J Atkinson RW Andersen ZJ Oftedal B Stafoggia M Lim YH. Long-term exposure to ambient air pollution and risk of lung cancer – a comparative analysis of incidence and mortality in four administrative cohorts in the ELAPSE study. Environ Res. (2024) 263:120236. doi: 10.1016/j.envres.2024.120236, PMID: 39455045

[29] Neupane BK Acharya BK Cao C Xu M Taylor PK Wang S. Lung cancer risk and its potential association with PM(2.5) in Bagmati province, Nepal-a spatiotemporal study from 2012 to 2021. Front Public Health. (2024) 12:1490973. doi: 10.3389/fpubh.2024.1490973, PMID: 39737461 PMC11683126

[30] Turnock ST Butt EW Richardson TB Mann GW Reddington CL Forster PM. The impact of European legislative and technology measures to reduce air pollutants on air quality, human health and climate. Environ Res Lett. (2016) 11:024010. doi: 10.1088/1748-9326/11/2/024010

[31] Khomenko S Cirach M Pereira-Barboza E Mueller N Barrera-Gómez J Rojas-Rueda D. Premature mortality due to air pollution in European cities: a health impact assessment. Lancet Planet Health. (2021) 5:e121–34. doi: 10.1016/s2542-5196(20)30272-2, PMID: 33482109

[32] Gu Y Henze DK Nawaz MO Cao H Wagner UJ. Sources of PM(2.5)-associated health risks in Europe and corresponding emission-induced changes during 2005-2015. Geohealth. (2023) 7:e2022GH000767. doi: 10.1029/2022gh000767, PMID: 36949891 PMC10027220

[33] Yang X Zhang T Zhang X Chu C Sang S. Global burden of lung cancer attributable to ambient fine particulate matter pollution in 204 countries and territories, 1990-2019. Environ Res. (2022) 204:112023. doi: 10.1016/j.envres.2021.112023, PMID: 34520750

[34] Li J Lu X Liu F Liang F Huang K Yang X. Chronic effects of high fine particulate matter exposure on lung Cancer in China. Am J Respir Crit Care Med. (2020) 202:1551–9. doi: 10.1164/rccm.202001-0002OC, PMID: 32614242 PMC8168622

[35] Liu L Wang Y Zhao Y. Air pollutant emissions caused by receiving international industrial transfer in southeast Asian developing countries from 1990 to 2018. Sci Total Environ. (2024) 921:171110. doi: 10.1016/j.scitotenv.2024.171110, PMID: 38395172

[36] To AH Ha DT Nguyen HM Vo DH. The impact of foreign direct investment on environment degradation: evidence from emerging markets in Asia. Int J Environ Res Public Health. (2019) 16:1636. doi: 10.3390/ijerph16091636, PMID: 31083372 PMC6539116

[37] Xiao Q Geng G Xue T Liu S Cai C He K. Tracking PM(2.5) and O(3) pollution and the related health burden in China 2013-2020. Environ Sci Technol. (2022) 56:6922–32. doi: 10.1021/acs.est.1c04548, PMID: 34941243

[38] Xue T Liu J Zhang Q Geng G Zheng Y Tong D. Rapid improvement of PM2.5 pollution and associated health benefits in China during 2013–2017. Sci China Earth Sci. (2019) 62:1847–56. doi: 10.1007/s11430-018-9348-2

[39] Puzzolo E Zerriffi H Carter E Clemens H Stokes H Jagger P. Supply considerations for scaling up clean cooking fuels for household energy in low- and middle-income countries. Geohealth. (2019) 3:370–90. doi: 10.1029/2019gh000208, PMID: 32159025 PMC7038875

[40] Local Burden of Disease Household Air Pollution Collaborators. Mapping development and health effects of cooking with solid fuels in low-income and middle-income countries, 2000-18: a geospatial modelling study. Lancet Glob Health. (2022) 10:e1395–411. doi: 10.1016/s2214-109x(22)00332-1 36113526 PMC9638039

[41] Raaschou-Nielsen O Andersen ZJ Beelen R Samoli E Stafoggia M Weinmayr G. Air pollution and lung cancer incidence in 17 European cohorts: prospective analyses from the European study of cohorts for air pollution effects (ESCAPE). Lancet Oncol. (2013) 14:813–22. doi: 10.1016/s1470-2045(13)70279-1, PMID: 23849838

[42] Hu J Dong H Dong Y Zhou R Teixeira W He X. Cancer burden attributable to risk factors, 1990-2019: a comparative risk assessment. iScience. (2024) 27:109430. doi: 10.1016/j.isci.2024.109430, PMID: 38550992 PMC10972825

[43] Arnold M Pandeya N Byrnes G Renehan PAG Stevens GA Ezzati PM. Global burden of cancer attributable to high body-mass index in 2012: a population-based study. Lancet Oncol. (2015) 16:36–46. doi: 10.1016/s1470-2045(14)71123-4, PMID: 25467404 PMC4314462

