# Supplementary material for: Spatial patterns and temporal trends of tracheal, bronchus, and lung cancer attributed to particulate matter pollution in Asia and its 34 countries and territories, 1990–2021
Source: Front Public Health. 2025 Jul 24;13:1602454. doi: 10.3389/fpubh.2025.1602454 (PMC12328408; doi:10.3389/fpubh.2025.1602454)
Supplement: Supplementary file 1 [file Data_Sheet_1.pdf]

Spatial patterns and temporal trends of tracheal, bronchus, and lung cancer attributed to particulate matter pollution in Asia and its 34 countries and territories, 1990 - 2021

Minxia Yang[1], Feng Xuan[2], * Liejiong Wang[2], Ying Lou[3], Shengjian Yu[2]

[1] Department of Radiology, Shaoxing People's Hospital, Shaoxing, Zhejiang Province, China.

[2] Department of Radiation Oncology, Zhuji Affiliated Hospital of Wenzhou Medical University, Shaoxing, Zhejiang Province, China.

[3] Department of Medical Oncology, Zhuji Affiliated Hospital of Wenzhou Medical University, Shaoxing, Zhejiang Province, China.

*** Correspondence:** Feng Xuan

Email: xfeng8901@outlook.com

Index

Table S1. Classification of 34 countries and territories into 5 Asia GBD Regions.

Table S2. The proportion of TBLC-related DALYs attributed to APMP and HAP by four world regions from 1990 to 2021.

Table S3. The proportion of TBLC-related DALYs attributed to APMP and HAP by five GBD regions from 1990 to 2021.

Table S4. DALYs and ASDR of TBLC attributed to APMP and HAP in 1990 and 2021 across 34 countries and territories, and the AAPC of ASDR from 1990 to 2021.

Table S5: The proportions of changed DALYs for TBLC attributed to APMP according to population aging, population growth, and epidemiological change between 1990 and 2021.

Table S6. The proportions of changed DALYs for TBLC attributed to HAP according to population aging, population growth, and epidemiological change between 1990 and 2021.

Table S7. DALYs and ASDR of TBLC attributed to APMP and HAP in different sex groups in 1990 and 2021 across four world regions and five GBD regions, and the AAPC of ASDR from 1990 to 2021.

Table S8. The proportion of TBLC-related DALYs attributed to APMP and HAP by four age groups across four world regions and five GBD regions in 1990 and 2021.

Table S9. Age-special DALY rates of TBLC attributed to APMP and HAP in different age groups in 2021, and their AAPC from 1990 to 2021 by four world regions and five GBD regions.

Figure S1. DALYs of TBLC attributed to APMP and HAP from 1990 t0 2021 across four world regions and five Asia GBD regions.

Figure S2. ASDR of TBLC attributed to APMP among 34 Asian countries and territories in 2021.

Figure S3. AAPC of ASDR of TBLC attributed to APMP among 34 Asian countries and territories from 1990 to 2021.

Figure S4. ASDR of TBLC attributed to HAP among 34 Asian countries and territories in 2021.

Figure S5. AAPC of ASDR of TBLC attributed to HAP among 34 Asian countries and territories from 1990 to 2021.

Table S1. Classification of 34 countries and territories into 5 Asia GBD Regions.

**Region**	**34 countries and territories**
High - income Asia Pacific	Republic of Korea; Japan; Singapore; Brunei Darussalam.
East Asia	China; Taiwan (Province of China); Democratic People's Republic of Korea.
Southeast Asia	Malaysia; Seychelles; Mauritius; Thailand; Indonesia; Uzbekistan; Philippines; Viet Nam; Myanmar; Timor - Leste; Lao People's Democratic Republic; Cambodia; Maldives.
Central Asia	Kazakhstan; Georgia; Armenia; Azerbaijan; Turkmenistan; Mongolia; Kyrgyzstan; Tajikistan.
South Asia	India; Bangladesh; Bhutan; Pakistan; Nepal.

Table S2. The proportion of TBLC-related DALYs attributed to APMP and HAP by four world regions from 1990 to 2021.

TBLC= Tracheal, bronchus, and lung cancer. DALYs = Disability-Adjusted Life Years. ASDR = Age-Standardized DALY Rate; APMP = Ambient Particulate Matter Pollution. HAP = Household Air Pollution.

Air pollution exposure	year	Asia	America	Europe	Africa
Ambient particulate matter pollution	1990	33.56	16.54	48.04	1.86
Ambient particulate matter pollution	1991	34.47	16.45	47.19	1.89
Ambient particulate matter pollution	1992	35.33	16.31	46.44	1.93
Ambient particulate matter pollution	1993	36.17	16.17	45.74	1.92
Ambient particulate matter pollution	1994	37.38	15.99	44.65	1.97
Ambient particulate matter pollution	1995	38.78	15.87	43.34	2.01
Ambient particulate matter pollution	1996	40.69	15.69	41.53	2.10
Ambient particulate matter pollution	1997	42.45	15.42	39.88	2.26
Ambient particulate matter pollution	1998	44.38	15.12	38.20	2.31
Ambient particulate matter pollution	1999	46.18	14.70	36.80	2.32
Ambient particulate matter pollution	2000	48.24	14.19	35.25	2.32
Ambient particulate matter pollution	2001	50.25	13.84	33.56	2.35
Ambient particulate matter pollution	2002	52.29	13.43	31.87	2.41
Ambient particulate matter pollution	2003	54.41	12.93	30.18	2.48
Ambient particulate matter pollution	2004	56.63	12.32	28.54	2.51
Ambient particulate matter pollution	2005	58.19	11.76	27.49	2.56
Ambient particulate matter pollution	2006	59.35	11.33	26.63	2.68
Ambient particulate matter pollution	2007	60.84	10.55	25.84	2.76
Ambient particulate matter pollution	2008	62.42	9.73	25.01	2.85
Ambient particulate matter pollution	2009	64.07	9.03	23.98	2.92

Air pollution exposure	year	Asia	America	Europe	Africa
Ambient particulate matter pollution	2010	65.94	8.26	22.86	2.93
Ambient particulate matter pollution	2011	67.93	7.74	21.42	2.92
Ambient particulate matter pollution	2012	69.99	7.23	19.88	2.91
Ambient particulate matter pollution	2013	71.90	6.80	18.42	2.88
Ambient particulate matter pollution	2014	73.35	6.46	17.27	2.91
Ambient particulate matter pollution	2015	74.61	6.14	16.29	2.97
Ambient particulate matter pollution	2016	75.58	5.91	15.54	2.97
Ambient particulate matter pollution	2017	76.51	5.64	14.89	2.97
Ambient particulate matter pollution	2018	77.22	5.42	14.33	3.03
Ambient particulate matter pollution	2019	77.87	5.33	13.72	3.07
Ambient particulate matter pollution	2020	77.87	5.80	13.15	3.17
Ambient particulate matter pollution	2021	78.73	5.22	12.94	3.11
Household air pollution	1990	89.20	2.41	4.51	3.89
Household air pollution	1991	89.13	2.32	4.63	3.91
Household air pollution	1992	89.08	2.29	4.69	3.94
Household air pollution	1993	89.07	2.22	4.74	3.97
Household air pollution	1994	89.07	2.16	4.79	3.98
Household air pollution	1995	89.09	2.10	4.83	3.98
Household air pollution	1996	89.19	2.03	4.76	4.02
Household air pollution	1997	89.23	1.98	4.74	4.05
Household air pollution	1998	89.39	1.93	4.59	4.10
Household air pollution	1999	89.55	1.87	4.52	4.07
Household air pollution	2000	89.74	1.82	4.41	4.04

Air pollution exposure	year	Asia	America	Europe	Africa
Household air pollution	2001	89.94	1.82	4.18	4.05
Household air pollution	2002	90.13	1.82	3.93	4.12
Household air pollution	2003	90.31	1.85	3.63	4.21
Household air pollution	2004	90.51	1.86	3.33	4.30
Household air pollution	2005	90.60	1.88	3.10	4.42
Household air pollution	2006	90.54	1.91	2.88	4.67
Household air pollution	2007	90.58	1.92	2.63	4.87
Household air pollution	2008	90.60	1.92	2.38	5.10
Household air pollution	2009	90.55	1.98	2.10	5.38
Household air pollution	2010	90.47	1.97	1.90	5.66
Household air pollution	2011	90.21	1.97	1.79	6.03
Household air pollution	2012	89.75	2.00	1.73	6.53
Household air pollution	2013	89.08	2.05	1.67	7.19
Household air pollution	2014	88.31	2.10	1.64	7.95
Household air pollution	2015	87.66	2.14	1.60	8.60
Household air pollution	2016	86.98	2.19	1.53	9.30
Household air pollution	2017	86.33	2.22	1.45	10.01
Household air pollution	2018	85.62	2.26	1.38	10.73
Household air pollution	2019	84.95	2.30	1.32	11.43
Household air pollution	2020	84.33	2.33	1.27	12.07
Household air pollution	2021	85.02	2.24	1.22	11.52

Table S3. The proportion of TBLC-related DALYs attributed to APMP and HAP by five GBD regions from 1990 to 2021.

TBLC= Tracheal, bronchus, and lung cancer. DALYs = Disability-Adjusted Life Years. ASDR = Age-Standardized DALY Rate; APMP = Ambient Particulate Matter Pollution. HAP = Household Air Pollution.

Air pollution exposure	year	High-income Asia Pacific	East Asia	Southeast Asia	Central Asia	South Asia
Ambient particulate matter pollution	1990	10.83	59.67	10.20	3.99	15.31
Ambient particulate matter pollution	1991	10.70	59.75	10.57	3.71	15.27
Ambient particulate matter pollution	1992	10.51	59.82	10.94	3.43	15.30
Ambient particulate matter pollution	1993	10.26	60.14	11.24	3.15	15.22
Ambient particulate matter pollution	1994	9.99	60.56	11.44	2.79	15.22
Ambient particulate matter pollution	1995	9.84	60.93	11.50	2.62	15.10
Ambient particulate matter pollution	1996	9.60	61.50	11.54	2.35	15.01
Ambient particulate matter pollution	1997	9.34	62.31	11.17	2.06	15.13
Ambient particulate matter pollution	1998	9.14	62.99	11.14	1.78	14.95
Ambient particulate matter pollution	1999	8.81	64.08	11.07	1.55	14.49
Ambient particulate matter pollution	2000	8.40	65.40	10.67	1.42	14.11
Ambient particulate matter pollution	2001	7.91	66.64	10.26	1.31	13.88
Ambient particulate matter pollution	2002	7.39	67.86	10.00	1.24	13.52
Ambient particulate matter pollution	2003	6.90	69.15	9.61	1.18	13.16
Ambient particulate matter pollution	2004	6.49	70.56	9.24	1.11	12.59
Ambient particulate matter pollution	2005	6.18	71.43	8.97	1.08	12.34
Ambient particulate matter pollution	2006	5.90	72.03	8.80	1.07	12.20
Ambient particulate matter pollution	2007	5.51	72.91	8.50	1.09	12.00
Ambient particulate matter pollution	2008	5.07	74.13	8.14	1.13	11.54
Ambient particulate matter pollution	2009	4.69	75.26	7.78	1.14	11.14

Air pollution exposure	year	High-income Asia Pacific	East Asia	Southeast Asia	Central Asia	South Asia
Ambient particulate matter pollution	2010	4.39	76.05	7.49	1.13	10.94
Ambient particulate matter pollution	2011	4.23	76.33	7.08	1.10	11.26
Ambient particulate matter pollution	2012	4.13	76.16	6.62	1.06	12.02
Ambient particulate matter pollution	2013	4.10	75.66	6.29	1.01	12.94
Ambient particulate matter pollution	2014	4.09	74.96	6.14	0.99	13.81
Ambient particulate matter pollution	2015	3.99	74.48	6.17	0.96	14.40
Ambient particulate matter pollution	2016	3.92	74.00	6.40	0.95	14.73
Ambient particulate matter pollution	2017	3.87	73.07	6.81	0.91	15.33
Ambient particulate matter pollution	2018	3.86	71.99	7.31	0.88	15.95
Ambient particulate matter pollution	2019	3.83	71.33	7.78	0.87	16.19
Ambient particulate matter pollution	2020	3.81	72.30	8.02	0.86	15.01
Ambient particulate matter pollution	2021	3.71	71.54	7.83	0.84	16.08
Household air pollution	1990	0.06	68.66	11.10	0.98	19.21
Household air pollution	1991	0.05	68.61	11.06	1.02	19.26
Household air pollution	1992	0.04	68.33	11.07	1.04	19.52
Household air pollution	1993	0.03	68.24	11.05	1.03	19.65
Household air pollution	1994	0.02	68.01	11.04	1.00	19.93
Household air pollution	1995	0.02	67.87	10.97	1.03	20.12
Household air pollution	1996	0.02	67.69	10.94	1.04	20.31
Household air pollution	1997	0.01	67.34	10.86	1.05	20.73
Household air pollution	1998	0.01	67.16	10.86	1.04	20.92
Household air pollution	1999	0.01	67.34	10.86	1.03	20.76
Household air pollution	2000	0.01	67.47	10.77	1.01	20.74

Air pollution exposure	year	High-income Asia Pacific	East Asia	Southeast Asia	Central Asia	South Asia
Household air pollution	2001	0.01	67.27	10.79	0.98	20.96
Household air pollution	2002	0.01	66.96	10.87	0.93	21.22
Household air pollution	2003	0.01	66.54	10.94	0.89	21.62
Household air pollution	2004	0.01	66.33	11.04	0.84	21.79
Household air pollution	2005	0.01	65.45	11.24	0.80	22.50
Household air pollution	2006	0.01	63.94	11.60	0.76	23.70
Household air pollution	2007	0.00	62.41	11.85	0.69	25.05
Household air pollution	2008	0.00	61.07	12.09	0.67	26.17
Household air pollution	2009	0.00	59.55	12.37	0.61	27.46
Household air pollution	2010	0.00	57.87	12.60	0.57	28.95
Household air pollution	2011	0.00	56.07	12.92	0.56	30.45
Household air pollution	2012	0.00	54.18	13.29	0.55	31.98
Household air pollution	2013	0.00	51.99	13.87	0.54	33.59
Household air pollution	2014	0.01	49.72	14.39	0.54	35.35
Household air pollution	2015	0.01	47.40	14.72	0.52	37.35
Household air pollution	2016	0.01	45.47	14.87	0.53	39.13
Household air pollution	2017	0.01	43.45	14.86	0.50	41.18
Household air pollution	2018	0.00	41.32	14.86	0.49	43.33
Household air pollution	2019	0.00	39.45	14.91	0.47	45.16
Household air pollution	2020	0.00	37.29	14.92	0.47	47.31
Household air pollution	2021	0.00	39.75	15.07	0.46	44.71

Table S4. DALYs and ASDR of TBLC attributed to APMP and HAP in 1990 and 2021 across 34 countries and territories, and the AAPC of ASDR from 1990 to 2021.

TBLC= Tracheal, bronchus, and lung cancer. DALYs = Disability-Adjusted Life Years. ASDR = Age-standardised DALYs rate. APMP = Ambient Particulate Matter Pollution. HAP = Household Air Pollution. AAPC = Average annual percentage change.

Location	Number in 1990 (95%CI)	Number in 2021 (95%CI)	ASDR in 1990 (per 100,000 population,95%CI)	ASDR in 2021 (per 100,000 population, 95%CI)	AAPC of ASDR (%, 95%CI)
**Ambient particulate matter pollution**					
Republic of Korea	38264(15099 to 70849)	81278(45033 to 124938)	212.77(84.09 to 393.61)	155.21(85.92 to 238.67)	–1.05(–1.32 to –0.78)
Japan	85139(15674 to 184544)	131631(62554 to 218376)	89.69(16.51 to 194.39)	67.37(32.19 to 111.36)	–0.97(–1.22 to –0.72)
Taiwan (Province of China)	16114(6441 to 30220)	31438(18897 to 45230)	172.14(68.77 to 323.07)	135.88(81.68 to 195.71)	–0.84(–1.39 to –0.28)
Singapore	3837(1347 to 6751)	3457(1681 to 5764)	306.39(107.61 to 538.38)	72.48(35.19 to 120.93)	–4.8(–5.82 to –3.76)
Brunei Darussalam	43(3 to 113)	86(16 to 183)	74.27(4.85 to 195.55)	42.91(8.21 to 90.86)	–1.75(–2.34 to –1.16)
Malaysia	7588(2942 to 13979)	16667(9015 to 26833)	143.42(55.74 to 263.48)	101.86(55.06 to 164.1)	–1.03(–1.55 to –0.5)
Seychelles	14(4 to 28)	24(7 to 49)	45.57(12.36 to 90.53)	35.11(10.8 to 72.54)	–1.1(–1.59 to –0.6)
Kazakhstan	20580(7057 to 38521)	11885(6965 to 17822)	271.27(92.84 to 507.96)	109.68(64.22 to 164.46)	–2.88(–3.38 to –2.38)
Mauritius	137(56 to 259)	258(81 to 486)	32.31(13.26 to 61.07)	24.76(7.76 to 46.59)	–0.97(–1.66 to –0.27)
Georgia	5128(1700 to 9916)	4433(2228 to 7053)	142.71(47.26 to 276.35)	140.76(70.86 to 223.97)	–0.32(–1.78 to 1.16)
Sri Lanka	1553(551 to 3347)	6996(2566 to 12890)	24.46(8.68 to 52.77)	45.15(16.56 to 83.17)	2.02(1.3 to 2.74)
Armenia	4488(1525 to 8280)	6237(3732 to 9028)	267.2(90.54 to 493.5)	257.46(153.94 to 372.98)	–0.23(–1.09 to 0.64)
Thailand	43601(17276 to 84091)	127243(71868 to 198576)	209.41(83.23 to 403.49)	213.87(120.84 to 334.21)	0.05(–0.25 to 0.35)
China	676321(295644 to 1262179)	4125753(2374169 to 5934144)	137.48(60.06 to 256.63)	345.81(199.13 to 497.72)	2.99(2.53 to 3.46)

Location	Number in 1990 (95%CI)	Number in 2021 (95%CI)	ASDR in 1990 (per 100,000 population,95%CI)	ASDR in 2021 (per 100,000 population, 95%CI)	AAPC of ASDR (%, 95%CI)
Azerbaijan	4143(1314 to 8726)	7150(3237 to 12831)	134.38(42.59 to 282.78)	110.4(49.9 to 198.17)	–0.77(–1.44 to –0.09)
Turkmenistan	1310(362 to 2570)	1599(797 to 2658)	111.71(30.8 to 218.97)	62.91(31.39 to 104.46)	–1.64(–2.37 to –0.91)
Indonesia	33747(12875 to 65401)	163693(76558 to 272433)	56.61(21.49 to 109.76)	114.25(53.28 to 190.25)	2.21(1.79 to 2.63)
Uzbekistan	7322(2598 to 14304)	11320(6417 to 17319)	108.35(38.48 to 211.77)	68.28(38.73 to 104.35)	–1.6(–2.38 to –0.82)
Philippines	16480(7152 to 29196)	45341(23669 to 72573)	91.95(39.7 to 163.59)	92.81(48.48 to 148.51)	–0.02(–0.34 to 0.3)
Viet Nam	6748(2662 to 14318)	62153(26753 to 113758)	29.24(11.53 to 61.98)	104.22(44.93 to 190.13)	4.24(4.05 to 4.43)
Mongolia	480(170 to 1080)	2624(1147 to 4220)	79.8(28.27 to 179.57)	186.68(81.62 to 299.29)	2.6(2.24 to 2.95)
Kyrgyzstan	2114(709 to 4487)	1668(753 to 2942)	121.21(40.62 to 257.52)	56.46(25.58 to 99.44)	–2.6(–3.17 to –2.02)
India	64585(32636 to 107065)	375887(219776 to 562794)	22.16(11.2 to 36.8)	53.5(31.24 to 80.12)	2.81(2.33 to 3.29)
Maldives	27(8 to 62)	51(23 to 90)	51.93(15.24 to 117.69)	26.3(12.1 to 45.85)	–2.32(–3.24 to –1.39)
Democratic People's Republic of Korea	9004(3207 to 19432)	11517(5478 to 21381)	91.59(32.81 to 197.03)	60.7(28.89 to 112.57)	–1.3(–1.46 to –1.14)
Tajikistan	1315(399 to 2818)	1775(864 to 3262)	80.73(24.52 to 172.93)	47.44(23.08 to 86.71)	–1.9(–2.46 to –1.34)
Myanmar	7931(3088 to 17355)	25814(10535 to 47777)	57.89(22.49 to 126.07)	91.24(37.31 to 168.65)	1.56(1.38 to 1.74)
Timor–Leste	40(12 to 96)	244(76 to 511)	23.61(6.87 to 56.01)	50(15.62 to 104.56)	2.32(1.88 to 2.77)
Lao People's Democratic Republic	636(219 to 1536)	2393(861 to 4703)	51.54(17.71 to 124.08)	87.93(31.73 to 172.22)	1.8(1.65 to 1.95)
Bangladesh	5757(2419 to 11302)	15703(7514 to 28969)	20.96(8.81 to 41.11)	19.74(9.46 to 36.38)	–0.17(–0.89 to 0.56)

Location	Number in 1990 (95%CI)	Number in 2021 (95%CI)	ASDR in 1990 (per 100,000 population,95%CI)	ASDR in 2021 (per 100,000 population, 95%CI)	AAPC of ASDR (%, 95%CI)
Cambodia	1287(445 to 2746)	4868(2026 to 9740)	48.41(16.66 to 103.35)	66.73(27.86 to 133.02)	1.31(1.02 to 1.6)
Bhutan	21(8 to 45)	149(69 to 257)	13.6(4.96 to 29.65)	43.07(19.89 to 73.95)	3.83(3 to 4.67)
Pakistan	18489(7933 to 33565)	72904(34810 to 126432)	57.15(24.51 to 103.72)	98.98(47.28 to 171.17)	1.62(1.09 to 2.14)
Nepal	1152(459 to 2379)	3838(1865 to 6633)	20.12(8 to 41.47)	28.36(13.81 to 48.89)	0.77(0.22 to 1.32)
**Household air pollution**					
Republic of Korea	1352(133 to 5883)	14(0 to 108)	7.55(0.74 to 32.7)	0.03(0 to 0.21)	–16.78(–17.09 to –16.47)
Japan	546(2 to 4808)	84(0 to 594)	0.58(0 to 5.07)	0.04(0 to 0.3)	–8.14(–8.37 to –7.9)
Taiwan (Province of China)	4123(110 to 21003)	236(0 to 1342)	44.08(1.18 to 224.49)	1.02(0 to 5.81)	–11.74(–12.05 to –11.43)
Singapore	58(0 to 630)	1(0 to 1)	4.65(0.01 to 50.38)	0.02(0 to 0.01)	–15.54(–16.93 to –14.12)
Brunei Darussalam	0(0 to 0)	0(0 to 0)	0.58(0 to 0.35)	0.11(0 to 0.12)	–5.41(–5.9 to –4.92)
Malaysia	513(61 to 1889)	44(0 to 367)	9.69(1.15 to 35.72)	0.27(0 to 2.25)	–11.36(–11.64 to –11.09)
Seychelles	2(0 to 9)	0(0 to 1)	5.38(0.18 to 27.45)	0.1(0 to 0.94)	–12.22(–13.12 to –11.31)
Kazakhstan	6648(497 to 24837)	198(0 to 1935)	87.91(6.6 to 327.8)	1.83(0 to 17.93)	–11.83(–12.47 to –11.19)
Mauritius	62(10 to 173)	3(0 to 26)	14.67(2.36 to 40.93)	0.28(0 to 2.47)	–12.24(–13.19 to –11.27)
Georgia	3564(443 to 10240)	1457(151 to 4874)	99.37(12.36 to 285.41)	46.26(4.79 to 154.66)	–2.91(–3.57 to –2.24)

Location	Number in 1990 (95%CI)	Number in 2021 (95%CI)	ASDR in 1990 (per 100,000 population,95%CI)	ASDR in 2021 (per 100,000 population, 95%CI)	AAPC of ASDR (%, 95%CI)
Sri Lanka	8245(4957 to 12385)	4865(408 to 16685)	129.68(78.16 to 194.01)	31.44(2.64 to 107.59)	–4.8(–5.35 to –4.26)
Armenia	2393(908 to 4686)	237(26 to 923)	142.94(54.25 to 279.7)	9.81(1.09 to 38.13)	–8.35(–9.49 to –7.19)
Thailand	59573(26497 to 102918)	8233(133 to 50925)	285.89(127.01 to 493.48)	13.85(0.22 to 85.67)	–9.43(–10.11 to –8.74)
China	2333082(1487108 to 3249300)	766682(106434 to 2596250)	471.53(300.76 to 655.77)	64.22(8.92 to 217.44)	–6.35(–6.77 to –5.93)
Azerbaijan	3436(600 to 8545)	285(4 to 1830)	111.95(19.6 to 278.18)	4.42(0.06 to 28.39)	–10.09(–10.87 to –9.31)
Turkmenistan	20(3 to 76)	1(0 to 11)	1.72(0.25 to 6.5)	0.05(0 to 0.42)	–10.83(–11.99 to –9.65)
Indonesia	121373(72846 to 176814)	96125(28632 to 223452)	205.65(123 to 299.08)	67.55(20.11 to 156.6)	–3.62(–3.76 to –3.49)
Uzbekistan	7772(3685 to 13249)	2049(341 to 6099)	115.28(54.69 to 196.34)	12.37(2.06 to 36.8)	–7.18(–7.74 to –6.61)
Philippines	32244(19241 to 48052)	45884(19669 to 85538)	184.64(110.17 to 274.69)	94.42(40.52 to 175.8)	–2.16(–2.44 to –1.88)
Viet Nam	90355(55225 to 134799)	79664(27803 to 156921)	391.76(239.48 to 583.96)	133.88(46.83 to 262.79)	–3.48(–3.64 to –3.32)
Mongolia	2542(1456 to 4037)	906(69 to 3323)	421.72(242.14 to 668.65)	64.68(4.89 to 236.63)	–6.03(–6.64 to –5.41)
Kyrgyzstan	4202(2052 to 7031)	2116(1032 to 3503)	241.54(117.96 to 404.06)	71.74(35.22 to 118.71)	–4.01(–4.32 to –3.7)
India	229684(146777 to 311698)	309380(156704 to 534949)	79.31(50.68 to 107.7)	43.87(22.17 to 75.94)	–1.94(–2.15 to –1.74)
Maldives	86(39 to 149)	5(0 to 37)	163.84(75.41 to 285.15)	2.77(0.04 to 18.82)	–12.65(–13.29 to –12)

Location	Number in 1990 (95%CI)	Number in 2021 (95%CI)	ASDR in 1990 (per 100,000 population,95%CI)	ASDR in 2021 (per 100,000 population, 95%CI)	AAPC of ASDR (%, 95%CI)
Democratic People's Republic of Korea	38758(21093 to 61389)	76163(40176 to 127904)	396.88(216.97 to 625.92)	402.4(212.35 to 675.63)	0.03(–0.02 to 0.09)
Tajikistan	3473(1758 to 5665)	2602(1232 to 4651)	213.59(108.53 to 348.21)	69.43(33.06 to 123.16)	–3.66(–4.2 to –3.13)
Myanmar	54166(30340 to 87290)	53980(23903 to 95705)	394.83(220.77 to 635.6)	190.85(84.67 to 337.53)	–2.37(–2.53 to –2.21)
Timor–Leste	361(195 to 577)	728(282 to 1324)	212.07(115.04 to 338.08)	148.86(57.51 to 270.57)	–1.13(–1.43 to –0.82)
Lao People's Democratic Republic	5251(2724 to 9120)	6381(2525 to 11747)	425.13(221.35 to 737.52)	233.79(92.62 to 428.03)	–1.93(–2.06 to –1.8)
Bangladesh	42223(24414 to 68820)	68222(35977 to 114481)	153.35(88.84 to 249.34)	85.63(45.22 to 143.26)	–1.87(–2.25 to –1.48)
Cambodia	11290(6594 to 17536)	23273(12532 to 37018)	423.5(247.39 to 657.26)	319.33(172.17 to 506.6)	–0.96(–1.1 to –0.83)
Bhutan	116(56 to 201)	45(8 to 138)	75.76(37.12 to 131.46)	13.09(2.2 to 39.64)	–5.56(–5.87 to –5.26)
Pakistan	54809(30948 to 81457)	86773(41409 to 152737)	169.64(95.74 to 252.29)	117.87(56.29 to 207.8)	–1.16(–1.34 to –0.97)
Nepal	5504(2936 to 9105)	9736(5131 to 16306)	96.02(51.08 to 159.04)	71.86(37.96 to 120.05)	–0.92(–1.14 to –0.71)

Table S5: The proportions of changed DALYs for lTBLC attributed to APMP according to population aging, population growth, and epidemiological change between 1990 and 2021.

TBLC= Tracheal, bronchus, and lung cancer. DALY = Disability-Adjusted Life Years. APMP = Ambient Particulate Matter Pollution.

Location	Air pollution exposure	Overall difference	Aging	Population	Epidemiological change
Asia	Ambient particulate matter pollution	4357426.87(388.91%)	740011.03 (66.05%)	1771777.9 (158.14%)	1845637.95 (164.73%)
America	Ambient particulate matter pollution	–188799.75(–34.19%)	114521.3 (20.74%)	301598.25 (54.62%)	–604919.3 (–109.55%)
Africa	Ambient particulate matter pollution	154320.52(248.53%)	–3613.62 (–5.82%)	108742.2 (175.12%)	49191.95 (79.22%)
Europe	Ambient particulate matter pollution	–703241.11(–43.85%)	191842.37 (11.96%)	252439.83 (15.74%)	–1147523.3 (–71.56%)
High-income Asia Pacific	Ambient particulate matter pollution	89168.67(70.06%)	73442.25 (57.7%)	47547.55 (37.36%)	–31821.12 (–25%)
East Asia	Ambient particulate matter pollution	3467268.75(494.31%)	783912.75 (111.76%)	1036699.3 (147.8%)	1646656.69 (234.75%)
South Asia	Ambient particulate matter pollution	378478.15(420.51%)	31067.57 (34.52%)	178404.7 (198.22%)	169005.88 (187.78%)
Central Asia	Ambient particulate matter pollution	1811.15(3.86%)	3842.68 (8.2%)	27012.55 (57.62%)	–29044.07 (–61.95%)
Southeast Asia	Ambient particulate matter pollution	336420.16(280.44%)	60716.88 (50.61%)	177482.74 (147.95%)	98220.54 (81.88%)

Table S6. The proportions of changed DALYs for TBLC attributed to HAP according to population aging, population growth, and epidemiological change between 1990 and 2021.

TBLC= Tracheal, bronchus, and lung cancer. DALY = Disability-Adjusted Life Years. HAP = Household Air Pollution.

Location	Air pollution exposure	Overall difference	Aging	Population	Epidemiological change
Asia	Household air pollution from solid fuels	–1465160.16(–46.73%)	669626.02 (21.36%)	1938428.79 (61.82%)	–4073214.97 (–129.9%)
America	Household air pollution from solid fuels	–40513.08(–47.89%)	14923.91 (17.64%)	43669.96 (51.63%)	–99106.95 (–117.16%)
Africa	Household air pollution from solid fuels	89812.51(65.76%)	–5138.76 (–3.76%)	164607.77 (120.53%)	–69656.5 (–51%)
Europe	Household air pollution from solid fuels	–134408.22(–84.88%)	11985.76 (7.57%)	19414.81 (12.26%)	–165808.79 (–104.71%)
High–income Asia Pacific	Household air pollution from solid fuels	–1857.96(–94.93%)	413.32 (21.12%)	371.81 (19%)	–2643.1 (–135.04%)
East Asia	Household air pollution from solid fuels	–1532881.65(–64.52%)	745612.28 (31.38%)	1093458.84 (46.02%)	–3371952.78 (–141.92%)
South Asia	Household air pollution from solid fuels	141821.73(42.67%)	53914.57 (16.22%)	330915.29 (99.57%)	–243008.13 (–73.12%)
Central Asia	Household air pollution from solid fuels	–24198.29(–71.07%)	2040.23 (5.99%)	14434.64 (42.39%)	–40673.17 (–119.45%)
Southeast Asia	Household air pollution from solid fuels	–64445.44(–16.78%)	96143.06 (25.03%)	296375.44 (77.17%)	–456963.94 (–118.98%)

Table S7. DALYs and ASDR of TBLC attributed to APMP and HAP in different sex groups in 1990 and 2021 across four world regions and five GBD regions, and the AAPC of ASDR from 1990 to 2021.

TBLC= Tracheal, bronchus, and lung cancer. DALYs = Disability-Adjusted Life Years. ASDR = Age-standardised DALYs rate. APMP = Ambient Particulate Matter Pollution. HAP = Household Air Pollution. AAPC = Average annual percentage change.

Location	Sex	ASDR in 1990 (per 100,000 population,95%CI)	ASDR in 2021 (per 100,000 population, 95%CI)	AAPC of ASDR (%, 95%CI)
**Ambient particulate matter pollution**				
**World regional levels**				
Asia	Female	45.32(23.97 to 77.19)	114.29(63.58 to 168.8)	2.97(2.66 to 3.28)
	Male	145.53(76.88 to 241.78)	276.25(160.94 to 397.68)	2.05(1.71 to 2.4)
America	Female	99.55(46.05 to 166.27)	38.63(21.37 to 59.13)	–2.99(–3.43 to –2.54)
	Male	242.23(120.17 to 397.16)	61.48(34.2 to 93.3)	–4.27(–4.74 to –3.8)
Africa	Female	12.64(7.48 to 19.16)	24.06(14.41 to 35.23)	2.03(1.58 to 2.49)
	Male	61.03(36.64 to 89.46)	89.99(55.73 to 128.78)	1.25(0.83 to 1.66)
Europe	Female	83.03(45.51 to 125.38)	54.65(31.77 to 79.5)	–1.52(–1.71 to –1.33)
	Male	548.38(310.81 to 825.65)	176.82(105.09 to 257.42)	–3.63(–3.91 to –3.34)
**GBD regional levels**				
High-income Asia Pacific	Female	53.65(13.98 to 110.01)	43.74(22.54 to 70.11)	–0.72(–1.04 to –0.4)
	Male	188.95(48.05 to 385.46)	139.28(72.12 to 219.66)	–1(–1.24 to –0.76)
East Asia	Female	69.03(30.58 to 132.24)	207.84(111.34 to 313.53)	3.6(3.39 to 3.81)
	Male	210.92(91.39 to 399.86)	480.26(269.52 to 708.72)	2.66(2.17 to 3.16)
South Asia	Female	7.62(3.77 to 13.3)	27.66(15.27 to 42.76)	4.22(3.69 to 4.75)
	Male	41.71(20.35 to 69.95)	80.46(45.84 to 123.42)	2.04(1.58 to 2.5)
Central Asia	Female	49.16(18.14 to 95.06)	38.04(22.31 to 56.22)	–0.91(–1.47 to –0.35)
	Male	325.19(123.04 to 608.93)	175.51(105.36 to 255.3)	–2.05(–2.59 to –1.5)

Location	Sex	ASDR in 1990 (per 100,000 population,95%CI)	ASDR in 2021 (per 100,000 population, 95%CI)	AAPC of ASDR (%, 95%CI)
Southeast Asia	Female	37.77(15.22 to 73.11)	68.78(35.07 to 107.88)	1.91(1.7 to 2.13)
	Male	127.19(54.18 to 234.36)	179.42(98.76 to 274.91)	1.08(0.87 to 1.28)
**Household air pollution**				
**World regional levels**				
Asia	Female	163.91(103.55 to 230.96)	41.06(14.5 to 99.24)	–4.46(–4.87 to –4.05)
	Male	358.45(223.63 to 500.15)	75.44(25.5 to 190.46)	–4.97(–5.27 to –4.67)
America	Female	16.53(7.26 to 32.15)	5.64(1.93 to 14.59)	–3.51(–3.84 to –3.18)
	Male	35.4(12.98 to 75.58)	6.65(2.17 to 18.55)	–5.3(–5.57 to –5.03)
Africa	Female	37.23(23.37 to 53.81)	34.29(20.4 to 50.13)	–0.3(–0.34 to –0.25)
	Male	124.71(78.29 to 184.44)	82.82(50.93 to 122.65)	–1.35(–1.48 to –1.22)
Europe	Female	9.91(2.29 to 29.42)	1.69(0.09 to 10.48)	–5.67(–5.94 to –5.4)
	Male	51.64(11.69 to 167.45)	4.52(0.3 to 26.88)	–7.62(–7.95 to –7.29)
**GBD regional levels**				
High-income Asia Pacific	Female	1.09(0.08 to 6.47)	0.03(0 to 0.18)	–11.57(–12.11 to –11.03)
	Male	2.5(0.16 to 15.18)	0.06(0 to 0.38)	–11.71(–12.14 to –11.28)
East Asia	Female	308.96(192.81 to 439.13)	51.2(10.3 to 159.69)	–5.77(–6.3 to –5.23)
	Male	627.5(387.02 to 897.06)	86.73(16.34 to 286.33)	–6.29(–6.74 to –5.83)
South Asia	Female	40.31(25.47 to 54.51)	35.17(18.93 to 57.14)	–0.46(–0.66 to –0.27)
	Male	143.56(89.63 to 199.29)	73.99(37.8 to 128.05)	–2.1(–2.37 to –1.84)
Central Asia	Female	51.38(20.1 to 102.96)	10.96(3.95 to 26.46)	–4.96(–5.49 to –4.42)
	Male	219.07(79.08 to 498.39)	31.13(9.89 to 81.88)	–6.23(–6.65 to –5.8)
Southeast Asia	Female	145.57(86.82 to 214.17)	56.15(22.84 to 112.55)	–3.07(–3.2 to –2.95)
	Male	379.68(230.09 to 542.43)	112.81(41.1 to 227.31)	–3.93(–4.1 to –3.77)

Table S8. The proportion of TBLC-related DALYs attributed to APMP and HAP by four age groups across four world regions and five GBD regions in 1990 and 2021.

TBLC= Tracheal, bronchus, and lung cancer. DALY = Disability-Adjusted Life Years. APMP = Ambient Particulate Matter Pollution. HAP = Household Air Pollution.

Location	Age group	DALYs in 1990 (95%UI)	DALYs in 2021 (95%UI)	Age group percentage in 1990	Age group percentage in 2021
**Ambient particulate matter pollution**					
**World regional levels**					
Asia	25 44 years	125423(64221 to 206705)	317654(186996 to 455727)	11.2	5.8
Asia	45 59 years	387943(202325 to 647073)	1558178(914375 to 2211257)	34.6	28.4
Asia	60 74 years	486316(256824 to 809099)	2544690(1494401 to 3583695)	43.4	46.5
Asia	75 plus years	120725(63495 to 199995)	1057313(628401 to 1505675)	10.8	19.3
America	25 44 years	33383(18084 to 52507)	16010(9586 to 23225)	6	4.4
America	45 59 years	157815(80830 to 256188)	88278(50274 to 132959)	28.6	24.3
America	60 74 years	273840(128977 to 457184)	179373(98946 to 274182)	49.6	49.4
America	75 plus years	87148(40502 to 143565)	79725(43078 to 123370)	15.8	21.9
Africa	25 44 years	7792(4660 to 11413)	21638(13347 to 31333)	12.5	10
Africa	45 59 years	23786(14469 to 34716)	79887(49307 to 115466)	38.3	36.9
Africa	60 74 years	25064(15184 to 36426)	93755(58067 to 134010)	40.4	43.3
Africa	75 plus years	5453(3308 to 8022)	21135(13265 to 30063)	8.8	9.8
Europe	25 44 years	88979(52087 to 131756)	26843(15699 to 39205)	5.5	3
Europe	45 59 years	558346(324747 to 836883)	224446(133980 to 326229)	34.8	24.9
Europe	60 74 years	751370(422645 to 1126323)	477466(284467 to 688836)	46.9	53
Europe	75 plus years	204980(108398 to 315207)	171679(99592 to 250589)	12.8	19.1
**GBD regional levels**					
High-income Asia Pacific	25 44 years	9069(2720 to 18079)	3676(1939 to 5699)	7.1	1.7

Location	Age group	DALYs in 1990 (95%UI)	DALYs in 2021 (95%UI)	Age group percentage in 1990	Age group percentage in 2021
High-income Asia Pacific	45 59 years	34753(9859 to 69281)	26560(14074 to 41318)	27.3	12.3
High-income Asia Pacific	60 74 years	57830(14914 to 117233)	90959(47520 to 142360)	45.4	42
High-income Asia Pacific	75 plus years	25630(5420 to 54517)	95257(47432 to 151884)	20.1	44
East Asia	25 44 years	84467(37586 to 156334)	212462(122841 to 309593)	12	5.1
East Asia	45 59 years	241175(105469 to 454585)	1146578(649756 to 1645471)	34.4	27.5
East Asia	60 74 years	304985(132980 to 565397)	1970100(1141145 to 2824495)	43.5	47.3
East Asia	75 plus years	70813(31149 to 133211)	839568(488845 to 1212205)	10.1	20.1
South Asia	25 44 years	21643(10913 to 35873)	98781(57093 to 150148)	12	10.5
South Asia	45 59 years	72935(36539 to 120120)	350907(201953 to 534842)	40.5	37.5
South Asia	60 74 years	73786(36062 to 124014)	404041(230743 to 613143)	41	43.1
South Asia	75 plus years	11645(5658 to 19521)	83236(47338 to 126999)	6.5	8.9
Central Asia	25 44 years	4495(1718 to 8413)	4068(2430 to 5901)	9.6	8.4
Central Asia	45 59 years	20867(7818 to 39109)	17431(10424 to 25572)	44.5	35.8
Central Asia	60 74 years	18881(7224 to 35702)	23627(14289 to 34341)	40.3	48.5
Central Asia	75 plus years	2638(998 to 5045)	3565(2144 to 5242)	5.6	7.3
Southeast Asia	25 44 years	12977(5617 to 23551)	34945(19072 to 53208)	10.8	7.7
Southeast Asia	45 59 years	44711(19298 to 81735)	152931(82255 to 234011)	37.3	33.5
Southeast Asia	60 74 years	51117(21700 to 94641)	210191(114372 to 320740)	42.6	46.1
Southeast Asia	75 plus years	11156(4925 to 20658)	58315(32644 to 87267)	9.3	12.8
**Household air pollution**					
**World regional levels**					
Asia	25 44 years	388134(250650 to 541062)	133718(54563 to 287188)	12.4	8

Location	Age group	DALYs in 1990 (95%UI)	DALYs in 2021 (95%UI)	Age group percentage in 1990	Age group percentage in 2021
Asia	45 59 years	1124578(721179 to 1553209)	557025(207294 to 1313399)	35.9	33.3
Asia	60 74 years	1334852(864826 to 1831242)	742714(254176 to 1861225)	42.6	44.5
Asia	75 plus years	288056(184763 to 395288)	237004(69081 to 654890)	9.2	14.2
America	25 44 years	9132(3738 to 18190)	3789(1422 to 8964)	10.8	8.6
America	45 59 years	28299(11041 to 58810)	13584(4900 to 34640)	33.5	30.8
America	60 74 years	36083(14118 to 75188)	19662(6694 to 53308)	42.7	44.6
America	75 plus years	11075(4401 to 22859)	7042(2060 to 20939)	13.1	16
Africa	25 44 years	13529(8379 to 19809)	25179(15152 to 37894)	9.9	11.1
Africa	45 59 years	52041(32712 to 76106)	85433(51549 to 128000)	38.1	37.7
Africa	60 74 years	60338(38912 to 86762)	94885(57743 to 137357)	44.2	41.9
Africa	75 plus years	10665(6767 to 15433)	20888(13172 to 29783)	7.8	9.2
Europe	25 44 years	11586(2592 to 36456)	797(56 to 4545)	7.3	3.3
Europe	45 59 years	64486(14709 to 202611)	6717(440 to 39328)	40.7	28
Europe	60 74 years	69025(15612 to 222537)	13253(836 to 83406)	43.6	55.3
Europe	75 plus years	13261(2866 to 44497)	3182(166 to 20395)	8.4	13.3
**GBD regional levels**					
High-income Asia Pacific	25 44 years	192(16 to 1019)	2(0 to 10)	9.8	1.5
High-income Asia Pacific	45 59 years	631(49 to 3517)	11(0 to 71)	32.2	10.7
High-income Asia Pacific	60 74 years	857(58 to 5357)	41(0 to 285)	43.8	41
High-income Asia Pacific	75 plus years	277(13 to 1980)	46(0 to 334)	14.1	46.7
East Asia	25 44 years	306101(194955 to 430538)	48378(10573 to 145461)	12.9	5.7
East Asia	45 59 years	836213(527412 to 1167745)	254327(55010 to 781836)	35.2	30.2

Location	Age group	DALYs in 1990 (95%UI)	DALYs in 2021 (95%UI)	Age group percentage in 1990	Age group percentage in 2021
East Asia	60 74 years	1004547(646604 to 1393132)	383245(72183 to 1238978)	42.3	45.5
East Asia	75 plus years	229102(147029 to 316387)	157131(26901 to 517692)	9.6	18.6
South Asia	25 44 years	80551(51220 to 109514)	109943(59462 to 177346)	12.1	11.6
South Asia	45 59 years	264479(168819 to 360493)	353229(186371 to 591738)	39.8	37.2
South Asia	60 74 years	275045(176233 to 374580)	398420(208192 to 662066)	41.4	42
South Asia	75 plus years	44596(28206 to 61117)	86723(44758 to 144534)	6.7	9.1
Central Asia	25 44 years	3482(1329 to 7530)	1060(394 to 2482)	10.2	10.8
Central Asia	45 59 years	14446(5193 to 32717)	3622(1252 to 9135)	42.4	36.8
Central Asia	60 74 years	13935(5146 to 30779)	4477(1533 to 11645)	40.9	45.4
Central Asia	75 plus years	2187(825 to 4751)	694(214 to 1900)	6.4	7
Southeast Asia	25 44 years	37282(22792 to 53185)	25450(9883 to 51080)	9.7	8
Southeast Asia	45 59 years	138968(84546 to 197761)	112234(41808 to 221167)	36.2	35.1
Southeast Asia	60 74 years	174416(109417 to 244711)	148290(56035 to 291600)	45.4	46.4
Southeast Asia	75 plus years	33410(20034 to 46642)	33657(12693 to 68646)	8.7	10.5

Table S9. Age-special DALY rates of TBLC attributed to APMP and HAP in different age groups in 2021, and their AAPC from 1990 to 2021 by four world regions and five GBD regions.

TBLC= Tracheal, bronchus, and lung cancer. DALY = Disability-Adjusted Life Years. APMP = Ambient Particulate Matter Pollution. HAP = Household Air Pollution.

Location	Age	Age-special DALY rates in 1990 (per 100,000 population,95%UI)	Age-special DALY rates in 2021 (per 100,000 population, 95%UI)	AAPC (%, 95%CI)
**Ambient Particulate Matter Pollution**				
**World regional levels**				
Asia	25 to 29	3.86(2.05 to 6.5)	5.74(3.32 to 8.23)	1.23(0.87 to 1.59)
Asia	30 to 34	8.31(4.29 to 13.86)	13.97(8.19 to 20)	1.63(1.02 to 2.24)
Asia	35 to 39	17.64(8.97 to 29.17)	25.83(15.22 to 36.78)	1.1(0.6 to 1.6)
Asia	40 to 44	34.96(17.82 to 57.04)	51.47(30.39 to 74.22)	1.18(0.56 to 1.81)
Asia	45 to 49	59.24(31.47 to 99.37)	96.57(56.31 to 138.62)	1.59(1.08 to 2.11)
Asia	50 to 54	112.23(58.79 to 187.17)	189.53(111.75 to 268.65)	1.66(1.3 to 2.02)
Asia	55 to 59	166.99(86.04 to 277.82)	296.97(174.08 to 419.89)	1.85(1.54 to 2.17)
Asia	60 to 64	221.88(117.74 to 365.77)	403.72(237.46 to 566.91)	1.9(1.52 to 2.28)
Asia	65 to 69	271.69(142.05 to 456.51)	571.06(333.71 to 803.14)	2.36(1.96 to 2.75)
Asia	70 to 74	297.79(158.24 to 495.55)	692.92(408.64 to 979.94)	2.75(2.46 to 3.04)
Asia	75 plus	252.62(132.77 to 415.01)	675.99(407.56 to 964.15)	3.16(2.71 to 3.62)
America	25 to 29	3.47(1.94 to 5.17)	1.74(1.06 to 2.56)	–2.21(–2.54 to –1.88)
America	30 to 34	7.91(4.51 to 12.1)	3.25(1.99 to 4.7)	–2.8(–3.18 to –2.42)
America	35 to 39	18.21(9.96 to 28.57)	5.88(3.53 to 8.55)	–3.59(–3.82 to –3.36)
America	40 to 44	43.07(22.83 to 68.7)	11.42(6.75 to 16.52)	–4.18(–4.5 to –3.85)
America	45 to 49	100.57(53.28 to 163.05)	23.85(13.83 to 35.31)	–4.49(–4.67 to –4.31)
America	50 to 54	188.71(98.59 to 305.77)	46.28(26.82 to 68.78)	–4.46(–4.84 to –4.08)

Location	Age	Age-special DALY rates in 1990 (per 100,000 population,95%UI)	Age-special DALY rates in 2021 (per 100,000 population, 95%UI)	AAPC (%, 95%CI)
America	55 to 59	304.9(151.53 to 495.89)	81.08(45.42 to 123.77)	–4.19(–4.67 to –3.71)
America	60 to 64	436.45(211.77 to 725.61)	118.96(66.48 to 181.13)	–4.06(–4.56 to –3.55)
America	65 to 69	524.11(244.88 to 876.4)	148.38(82.1 to 225.64)	–3.92(–4.28 to –3.57)
America	70 to 74	540.14(247.54 to 904.61)	171.47(92.95 to 264.67)	–3.58(–4.13 to –3.02)
America	75 plus	396.54(183.55 to 650.55)	160.9(87.22 to 246.76)	–2.82(–3.38 to –2.26)
Africa	25 to 29	1.4(0.83 to 2.1)	1.78(1.1 to 2.65)	0.72(0.55 to 0.9)
Africa	30 to 34	3.26(1.97 to 4.84)	3.88(2.37 to 5.71)	0.52(0.16 to 0.88)
Africa	35 to 39	7.18(4.3 to 10.71)	7.38(4.51 to 10.52)	0.02(–0.46 to 0.51)
Africa	40 to 44	14.38(8.59 to 20.63)	15.07(9.38 to 21.79)	0.08(–0.23 to 0.38)
Africa	45 to 49	27.47(16.73 to 40.21)	31.56(19.43 to 45.27)	0.46(0.01 to 0.92)
Africa	50 to 54	48.82(29.5 to 72.27)	64.02(39.53 to 95)	0.85(0.58 to 1.13)
Africa	55 to 59	68.77(42.04 to 98.97)	102.05(63.05 to 144.94)	1.21(0.8 to 1.63)
Africa	60 to 64	89.04(54.21 to 128.77)	141.53(87.18 to 204.58)	1.49(1.06 to 1.93)
Africa	65 to 69	100.64(60.72 to 146.74)	172.11(106.18 to 247.38)	1.71(1.36 to 2.06)
Africa	70 to 74	102.07(61.69 to 148.84)	180.55(113.47 to 251.11)	1.82(1.51 to 2.13)
Africa	75 plus	86.69(52.27 to 129.08)	145.14(92.52 to 204.59)	1.68(1.37 to 1.99)
Europe	25 to 29	6.06(3.51 to 8.91)	2.48(1.49 to 3.59)	–3.03(–3.6 to –2.46)
Europe	30 to 34	15.47(8.83 to 22.75)	4.95(2.91 to 7.2)	–3.8(–4.21 to –3.39)
Europe	35 to 39	40.62(23.81 to 60.64)	11.12(6.57 to 16.45)	–4.25(–4.57 to –3.93)
Europe	40 to 44	99.84(58.72 to 147.54)	26.25(15.24 to 38.18)	–4.4(–4.68 to –4.12)
Europe	45 to 49	226.6(131.45 to 342.03)	60.14(36.21 to 87.22)	–4.28(–4.53 to –4.03)
Europe	50 to 54	412.18(239.42 to 617.11)	121.2(72.81 to 178.17)	–3.94(–4.31 to –3.58)

Location	Age	Age-special DALY rates in 1990 (per 100,000 population,95%UI)	Age-special DALY rates in 2021 (per 100,000 population, 95%UI)	AAPC (%, 95%CI)
Europe	55 to 59	615.09(358.45 to 920.3)	207.47(123.06 to 299.73)	–3.57(–3.89 to –3.24)
Europe	60 to 64	791.5(454.73 to 1179.55)	303.19(181.83 to 436.67)	–3.15(–3.46 to –2.85)
Europe	65 to 69	803.1(450.03 to 1205.91)	365.77(218.09 to 527.36)	–2.6(–2.83 to –2.37)
Europe	70 to 74	732.14(395.6 to 1108.08)	355.09(209.72 to 513.7)	–2.35(–2.65 to –2.04)
Europe	75 plus	499.94(267.03 to 770.57)	254.85(146.71 to 372.14)	–2.17(–2.4 to –1.95)
**GBD regional levels**				
High-income Asia Pacific	25 to 29	4.5(1.52 to 8.48)	1.83(0.91 to 2.97)	–2.77(–3.53 to –2.01)
High-income Asia Pacific	30 to 34	10.42(3.56 to 20.11)	4.04(2.11 to 6.43)	–3.17(–3.47 to –2.87)
High-income Asia Pacific	35 to 39	19.24(5.76 to 38.38)	8.08(4.26 to 12.54)	–2.88(–3.22 to –2.53)
High-income Asia Pacific	40 to 44	34.27(9.73 to 69.38)	16.15(8.58 to 24.8)	–2.49(–2.77 to –2.2)
High-income Asia Pacific	45 to 49	61.61(17.19 to 124.23)	31.6(16.45 to 49.45)	–2.1(–2.65 to –1.54)
High-income Asia Pacific	50 to 54	108.17(31.48 to 215.11)	59.12(31.47 to 91.48)	–1.94(–2.24 to –1.64)
High-income Asia Pacific	55 to 59	171.49(48.14 to 340.73)	108.12(57.47 to 168.41)	–1.52(–1.94 to –1.1)
High-income Asia Pacific	60 to 64	249.49(66.59 to 499.65)	180.73(94.13 to 279.28)	–1.15(–1.47 to –0.84)
High-income Asia Pacific	65 to 69	328.82(84.47 to 660.38)	265.02(141.68 to 413.88)	–0.77(–1.03 to –0.5)
High-income Asia Pacific	70 to 74	392.24(97.59 to 814.52)	329.47(169.43 to 520.03)	–0.66(–0.98 to –0.34)
High-income Asia Pacific	75 plus	379.14(80.13 to 804.02)	416.69(207.35 to 668.06)	0.32(0.09 to 0.55)
East Asia	25 to 29	5.7(2.51 to 10.74)	12.82(7.24 to 18.36)	2.59(1.94 to 3.25)
East Asia	30 to 34	13.34(5.97 to 24.81)	29.05(16.96 to 42.38)	2.52(1.83 to 3.22)
East Asia	35 to 39	27.84(12.74 to 52.16)	55.46(32.42 to 79.69)	2.23(1.8 to 2.67)
East Asia	40 to 44	56.93(24.86 to 104.04)	108.75(62.42 to 160.01)	2.15(1.7 to 2.6)
East Asia	45 to 49	90.07(39.35 to 169.76)	172.3(97.56 to 249.08)	2.03(1 to 3.07)

Location	Age	Age-special DALY rates in 1990 (per 100,000 population,95%UI)	Age-special DALY rates in 2021 (per 100,000 population, 95%UI)	AAPC (%, 95%CI)
East Asia	50 to 54	165.65(72.49 to 314.59)	320.28(182.83 to 461.56)	2.12(1.76 to 2.48)
East Asia	55 to 59	245.19(107.22 to 459.54)	485.23(273.59 to 692.42)	2.13(1.61 to 2.66)
East Asia	60 to 64	314.05(138.8 to 583.07)	722.32(419.07 to 1040.08)	2.72(2.4 to 3.04)
East Asia	65 to 69	380.51(163.34 to 702.29)	949.76(549.95 to 1359.4)	2.97(2.39 to 3.55)
East Asia	70 to 74	420.46(183.55 to 782.37)	1218.21(704.95 to 1743.53)	3.46(3.06 to 3.86)
East Asia	75 plus	364.2(160.36 to 686.38)	1223.78(713.3 to 1750.36)	3.95(3.59 to 4.32)
South Asia	25 to 29	1.31(0.66 to 2.17)	2.52(1.44 to 3.78)	2.07(1.24 to 2.9)
South Asia	30 to 34	2.36(1.18 to 3.89)	5.15(2.95 to 7.8)	2.4(1.62 to 3.18)
South Asia	35 to 39	4.67(2.34 to 7.79)	9.73(5.56 to 14.91)	2.31(1.17 to 3.45)
South Asia	40 to 44	9.31(4.72 to 15.39)	21.18(12.37 to 32.18)	2.66(1.98 to 3.35)
South Asia	45 to 49	19.87(9.76 to 32.51)	41.32(24.09 to 62.96)	2.33(1.82 to 2.84)
South Asia	50 to 54	35.44(17.91 to 58.49)	69.46(39.92 to 106.06)	2.03(1.58 to 2.49)
South Asia	55 to 59	49.11(24.7 to 81.03)	108.05(61.81 to 164.48)	2.59(2.06 to 3.12)
South Asia	60 to 64	65.06(32.09 to 108.1)	132.56(75.37 to 200.65)	2.31(1.73 to 2.89)
South Asia	65 to 69	78.14(38.25 to 131.94)	157.24(90.01 to 235.93)	2.29(1.79 to 2.79)
South Asia	70 to 74	70.97(33.97 to 121.09)	153.73(88.1 to 237.94)	2.48(1.79 to 3.17)
South Asia	75 plus	47.33(23.07 to 79.12)	104.21(59.59 to 158.22)	2.42(1.63 to 3.22)
Central Asia	25 to 29	7.67(2.86 to 14.47)	4.97(2.89 to 7.3)	–1.69(–2.09 to –1.3)
Central Asia	30 to 34	15.57(5.95 to 29.1)	9.37(5.55 to 13.53)	–1.8(–2.54 to –1.05)
Central Asia	35 to 39	30.72(11.92 to 57.46)	15.56(9.18 to 22.44)	–2.26(–3.12 to –1.38)
Central Asia	40 to 44	72.54(27.62 to 135.62)	29.3(17.81 to 42.61)	–2.95(–3.64 to –2.25)
Central Asia	45 to 49	148.28(55.33 to 278.9)	61.01(36.37 to 89.85)	–2.88(–3.75 to –2.01)

Location	Age	Age-special DALY rates in 1990 (per 100,000 population,95%UI)	Age-special DALY rates in 2021 (per 100,000 population, 95%UI)	AAPC (%, 95%CI)
Central Asia	50 to 54	289.63(107.89 to 540.08)	113.05(67.77 to 165.85)	–3.14(–3.71 to –2.58)
Central Asia	55 to 59	382.03(144.16 to 718.56)	187.36(112.01 to 274.44)	–2.35(–2.9 to –1.79)
Central Asia	60 to 64	482.72(184.33 to 914.14)	272.91(164.77 to 397.04)	–1.97(–2.43 to –1.52)
Central Asia	65 to 69	440.45(170.05 to 830.92)	311.89(189.79 to 453.57)	–1.29(–2 to –0.58)
Central Asia	70 to 74	358.41(135.77 to 677.01)	333.37(200.3 to 483.1)	–0.18(–0.68 to 0.33)
Central Asia	75 plus	180.85(68.48 to 346.59)	183.97(112.02 to 272.25)	0.01(–0.89 to 0.91)
Southeast Asia	25 to 29	3.38(1.47 to 6.25)	5.12(2.86 to 7.76)	1.45(0.82 to 2.08)
Southeast Asia	30 to 34	6.35(2.75 to 11.62)	9.43(5.15 to 14.32)	1.28(0.73 to 1.83)
Southeast Asia	35 to 39	12.49(5.33 to 22.87)	17.41(9.69 to 26.3)	1.05(0.6 to 1.5)
Southeast Asia	40 to 44	27.18(11.86 to 48.66)	36.1(19.43 to 55.27)	0.89(0.63 to 1.15)
Southeast Asia	45 to 49	54.34(23.17 to 99.76)	71.84(37.87 to 109.8)	0.91(0.68 to 1.14)
Southeast Asia	50 to 54	99.5(42.47 to 178.98)	129.55(67.99 to 198.81)	0.84(0.57 to 1.11)
Southeast Asia	55 to 59	145.05(63.56 to 268.07)	197.54(109.18 to 301.76)	0.95(0.7 to 1.2)
Southeast Asia	60 to 64	201.03(85.19 to 378.14)	285.26(152.99 to 436.46)	1.12(0.88 to 1.35)
Southeast Asia	65 to 69	233.38(98.78 to 426.83)	358.04(195 to 552.03)	1.36(1.18 to 1.54)
Southeast Asia	70 to 74	240.78(102.97 to 441.21)	392.21(217.83 to 587.22)	1.57(1.42 to 1.73)
Southeast Asia	75 plus	189.24(84.3 to 343.4)	353.47(200.97 to 519.72)	1.99(1.75 to 2.23)
**Household Air Pollution**				
**World regional levels**				
Asia	25 to 29	12.28(8.04 to 16.95)	3.05(1.37 to 5.92)	–4.57(–5.24 to –3.9)
Asia	30 to 34	25.48(16.62 to 35.69)	5.79(2.35 to 12.48)	–4.81(–5.83 to –3.78)
Asia	35 to 39	55.32(36.16 to 76.68)	10.43(4.2 to 22.79)	–5.44(–5.89 to –4.98)

Location	Age	Age-special DALY rates in 1990 (per 100,000 population,95%UI)	Age-special DALY rates in 2021 (per 100,000 population, 95%UI)	AAPC (%, 95%CI)
Asia	40 to 44	107.03(68.18 to 149.82)	21.55(8.73 to 46.51)	–5.09(–5.38 to –4.79)
Asia	45 to 49	173.31(110.98 to 239.23)	38.32(15.11 to 83.9)	–4.85(–5.38 to –4.32)
Asia	50 to 54	327(209.92 to 452.68)	68.14(25.29 to 160.38)	–4.99(–5.34 to –4.63)
Asia	55 to 59	480.09(307.83 to 662.05)	101.14(36.69 to 246.58)	–4.99(–5.51 to –4.47)
Asia	60 to 64	617.16(394.76 to 850.19)	133.55(49.58 to 317.36)	–4.89(–5.1 to –4.69)
Asia	65 to 69	749.36(488.82 to 1029.28)	164.5(54.52 to 416.7)	–4.84(–5.27 to –4.41)
Asia	70 to 74	795.91(520.88 to 1083.06)	180.72(58.25 to 473.71)	–4.74(–5.03 to –4.45)
Asia	75 plus	602.77(386.98 to 825.18)	151.53(44.41 to 421.48)	–4.44(–4.91 to –3.96)
America	25 to 29	1.59(0.66 to 3.07)	0.43(0.15 to 1.09)	–4.22(–4.71 to –3.71)
America	30 to 34	2.81(1.2 to 5.54)	0.8(0.3 to 1.89)	–4.12(–4.73 to –3.52)
America	35 to 39	5.09(2.09 to 10.16)	1.4(0.52 to 3.32)	–4.17(–4.49 to –3.84)
America	40 to 44	9.85(3.96 to 19.85)	2.65(1.02 to 6.16)	–4.18(–4.45 to –3.9)
America	45 to 49	20.45(8.22 to 42.34)	4.85(1.77 to 11.6)	–4.56(–4.86 to –4.27)
America	50 to 54	35.39(13.77 to 72.58)	7.55(2.79 to 18.99)	–4.91(–5.2 to –4.63)
America	55 to 59	49.58(19.06 to 104.35)	10.73(3.78 to 28.48)	–4.85(–5.19 to –4.5)
America	60 to 64	62.76(24.26 to 132.02)	13.9(4.8 to 36.97)	–4.77(–5.03 to –4.52)
America	65 to 69	66.22(26.02 to 137.88)	16.53(5.72 to 44.65)	–4.39(–4.72 to –4.07)
America	70 to 74	66.89(26.47 to 137.64)	17.14(5.61 to 47.79)	–4.32(–4.5 to –4.14)
America	75 plus	50.4(20.1 to 103.64)	14.21(4.11 to 42.22)	–4.04(–4.37 to –3.7)
Africa	25 to 29	2.13(1.34 to 3.04)	1.76(1.02 to 2.68)	–0.64(–0.77 to –0.51)
Africa	30 to 34	5.21(3.17 to 7.68)	4.25(2.54 to 6.42)	–0.7(–0.87 to –0.53)
Africa	35 to 39	11.68(7.37 to 17.02)	8.48(5.14 to 12.57)	–1.07(–1.2 to –0.93)

Location	Age	Age-special DALY rates in 1990 (per 100,000 population,95%UI)	Age-special DALY rates in 2021 (per 100,000 population, 95%UI)	AAPC (%, 95%CI)
Africa	40 to 44	27.29(16.78 to 40.12)	18.53(11.2 to 28.03)	–1.27(–1.44 to –1.1)
Africa	45 to 49	56.17(35.59 to 82.41)	37.51(22.63 to 55.68)	–1.33(–1.46 to –1.2)
Africa	50 to 54	105.27(66.9 to 155.36)	68.81(41.88 to 104.26)	–1.41(–1.61 to –1.21)
Africa	55 to 59	158.14(98.11 to 229.15)	102.83(61.61 to 153.42)	–1.41(–1.63 to –1.19)
Africa	60 to 64	204.52(132.34 to 294.82)	136.23(80.67 to 200.61)	–1.35(–1.43 to –1.26)
Africa	65 to 69	250.91(160.07 to 358.15)	181.06(111.36 to 260.62)	–1.05(–1.19 to –0.91)
Africa	70 to 74	251.79(164.03 to 364.41)	187.01(116.73 to 265.8)	–0.98(–1.15 to –0.8)
Africa	75 plus	169.55(108.16 to 243.98)	143.44(90.62 to 203.16)	–0.57(–0.67 to –0.48)
Europe	25 to 29	0.97(0.24 to 2.97)	0.07(0 to 0.36)	–8.43(–9.18 to –7.68)
Europe	30 to 34	2.31(0.56 to 7.15)	0.15(0.01 to 0.8)	–8.7(–9.41 to –7.98)
Europe	35 to 39	5.57(1.28 to 16.99)	0.32(0.02 to 1.86)	–8.91(–9.4 to –8.42)
Europe	40 to 44	12.11(2.6 to 38.93)	0.79(0.05 to 4.55)	–8.56(–8.95 to –8.17)
Europe	45 to 49	26.41(5.83 to 82.71)	1.84(0.12 to 10.71)	–8.3(–8.79 to –7.81)
Europe	50 to 54	47.41(10.69 to 148.4)	3.66(0.23 to 21.34)	–8.13(–8.85 to –7.41)
Europe	55 to 59	71.03(16.53 to 224.04)	6.13(0.41 to 36.08)	–7.67(–8.07 to –7.27)
Europe	60 to 64	80.16(18.5 to 257.07)	8.99(0.61 to 55.28)	–7.07(–7.51 to –6.62)
Europe	65 to 69	71.77(15.97 to 230.56)	10.44(0.63 to 65.59)	–6.1(–6.5 to –5.69)
Europe	70 to 74	55.59(12.24 to 183.25)	8.74(0.51 to 56.84)	–5.85(–6.12 to –5.58)
Europe	75 plus	32.34(7 to 109.07)	4.72(0.25 to 30.28)	–6.09(–6.33 to –5.84)
**GBD regional levels**				
High-income Asia Pacific	25 to 29	0.13(0.01 to 0.65)	0(0 to 0)	–15.57(–16.54 to –14.6)
High-income Asia Pacific	30 to 34	0.28(0.02 to 1.37)	0(0 to 0.01)	–15.36(–16.27 to –14.44)

Location	Age	Age-special DALY rates in 1990 (per 100,000 population,95%UI)	Age-special DALY rates in 2021 (per 100,000 population, 95%UI)	AAPC (%, 95%CI)
High-income Asia Pacific	35 to 39	0.41(0.03 to 2.19)	0(0 to 0.02)	–14.55(–15.2 to –13.91)
High-income Asia Pacific	40 to 44	0.64(0.05 to 3.51)	0.01(0 to 0.04)	–13.66(–14.05 to –13.28)
High-income Asia Pacific	45 to 49	1.11(0.09 to 6.21)	0.01(0 to 0.09)	–13.41(–13.87 to –12.94)
High-income Asia Pacific	50 to 54	2.1(0.17 to 11.16)	0.02(0 to 0.16)	–13.6(–14.3 to –12.9)
High-income Asia Pacific	55 to 59	2.98(0.22 to 17.16)	0.04(0 to 0.28)	–12.99(–13.42 to –12.55)
High-income Asia Pacific	60 to 64	3.81(0.27 to 23.3)	0.07(0 to 0.48)	–12.21(–12.62 to –11.81)
High-income Asia Pacific	65 to 69	5.08(0.34 to 32.06)	0.12(0 to 0.81)	–11.57(–12.08 to –11.05)
High-income Asia Pacific	70 to 74	5.34(0.34 to 33.82)	0.16(0 to 1.13)	–10.86(–11.38 to –10.34)
High-income Asia Pacific	75 plus	4.1(0.2 to 29.35)	0.2(0 to 1.46)	–9.46(–9.97 to –8.95)
East Asia	25 to 29	21.5(13.74 to 30.02)	3.21(0.8 to 9.22)	–6.22(–7.02 to –5.42)
East Asia	30 to 34	48.2(30.78 to 68.08)	6.51(1.36 to 19.91)	–6.54(–7.36 to –5.71)
East Asia	35 to 39	100.71(65.4 to 143.04)	12.28(2.63 to 37.42)	–6.69(–7.2 to –6.17)
East Asia	40 to 44	205.36(128.9 to 286.93)	25.03(5.52 to 74.65)	–6.68(–7.3 to –6.06)
East Asia	45 to 49	319.16(199.68 to 441.44)	40.39(9.19 to 119.73)	–6.53(–7.13 to –5.93)
East Asia	50 to 54	581.6(364.47 to 814.41)	72.43(15.89 to 222.66)	–6.6(–7.01 to –6.2)
East Asia	55 to 59	833.88(530.49 to 1167.11)	103.92(21.77 to 323.93)	–6.62(–7.09 to –6.14)
East Asia	60 to 64	1044.72(668.47 to 1453.29)	145.87(30.64 to 468.59)	–6.25(–6.75 to –5.75)
East Asia	65 to 69	1250.96(802.75 to 1737.32)	185.35(34.35 to 594.91)	–6.08(–6.63 to –5.52)
East Asia	70 to 74	1368.82(892.19 to 1886.35)	228.72(39.51 to 749.74)	–5.71(–6.09 to –5.32)
East Asia	75 plus	1178.31(757.19 to 1617.23)	229.04(39.7 to 770.37)	–5.24(–5.88 to –4.6)
South Asia	25 to 29	4.99(3.07 to 6.87)	3.06(1.67 to 4.82)	–1.6(–2.09 to –1.1)
South Asia	30 to 34	9.02(5.68 to 12.25)	5.95(3.24 to 9.27)	–1.32(–1.7 to –0.95)

Location	Age	Age-special DALY rates in 1990 (per 100,000 population,95%UI)	Age-special DALY rates in 2021 (per 100,000 population, 95%UI)	AAPC (%, 95%CI)
South Asia	35 to 39	17.24(10.82 to 23.72)	10.84(5.94 to 17.83)	–1.6(–2.1 to –1.09)
South Asia	40 to 44	34.28(22.22 to 46.14)	22.93(12.28 to 37.16)	–1.31(–1.73 to –0.89)
South Asia	45 to 49	70.18(43.98 to 95.7)	41.19(21.93 to 67.83)	–1.74(–2.02 to –1.46)
South Asia	50 to 54	129.05(83.03 to 174.5)	70.88(37.84 to 118.72)	–1.93(–2.17 to –1.69)
South Asia	55 to 59	180.14(115.4 to 247.15)	108.19(56.26 to 182.92)	–1.66(–2 to –1.32)
South Asia	60 to 64	244.71(158.38 to 330.57)	129.52(67.16 to 219.1)	–2.04(–2.46 to –1.61)
South Asia	65 to 69	285.96(181.84 to 393.87)	151.92(78.92 to 250.02)	–1.99(–2.39 to –1.59)
South Asia	70 to 74	267.86(170.29 to 363.95)	158.06(84.16 to 259.22)	–1.67(–2.11 to –1.23)
South Asia	75 plus	181.27(116.02 to 246.38)	108.57(55.97 to 179.77)	–1.61(–1.88 to –1.34)
Central Asia	25 to 29	6.78(2.71 to 13.91)	1.49(0.58 to 3.31)	–4.93(–5.55 to –4.31)
Central Asia	30 to 34	12.87(5.08 to 26.88)	2.7(1.03 to 6.01)	–4.99(–5.66 to –4.31)
Central Asia	35 to 39	23.89(9.21 to 51.14)	4.12(1.52 to 9.64)	–5.61(–6.03 to –5.2)
Central Asia	40 to 44	52.37(19.17 to 117.82)	6.94(2.52 to 16.92)	–6.38(–6.8 to –5.95)
Central Asia	45 to 49	104.68(38.54 to 233.19)	13.78(4.84 to 34.35)	–6.32(–6.96 to –5.67)
Central Asia	50 to 54	191.96(67.11 to 446.12)	24.05(8.43 to 60.31)	–6.6(–7.09 to –6.1)
Central Asia	55 to 59	273.46(99.83 to 608.58)	37.07(12.62 to 94.36)	–6.31(–6.91 to –5.71)
Central Asia	60 to 64	338.19(122.19 to 761.62)	51.83(17.8 to 134.56)	–6.02(–6.72 to –5.32)
Central Asia	65 to 69	342.02(128.97 to 737.95)	59.03(20.3 to 153.65)	–5.83(–6.72 to –4.94)
Central Asia	70 to 74	287.94(109.53 to 622.92)	62.96(21.28 to 164.32)	–4.86(–5.59 to –4.12)
Central Asia	75 plus	149.93(56.64 to 325.37)	35.81(11.11 to 96.47)	–4.9(–5.38 to –4.41)
Southeast Asia	25 to 29	9.93(6.04 to 14.32)	4.02(1.67 to 7.87)	–2.96(–3.3 to –2.62)
Southeast Asia	30 to 34	18.4(11.41 to 26.77)	7.04(2.79 to 13.79)	–3.11(–3.37 to –2.85)

Location	Age	Age-special DALY rates in 1990 (per 100,000 population,95%UI)	Age-special DALY rates in 2021 (per 100,000 population, 95%UI)	AAPC (%, 95%CI)
Southeast Asia	35 to 39	36.09(21.96 to 51.43)	12.52(4.82 to 25.07)	–3.42(–3.59 to –3.26)
Southeast Asia	40 to 44	77.13(47.08 to 108.98)	25.94(9.93 to 52.73)	–3.53(–3.68 to –3.37)
Southeast Asia	45 to 49	158.58(95.95 to 226.84)	51.8(20.21 to 103.31)	–3.57(–3.72 to –3.42)
Southeast Asia	50 to 54	300.33(181.42 to 426.25)	94.77(34.84 to 187.2)	–3.73(–3.86 to –3.6)
Southeast Asia	55 to 59	475.53(291.57 to 676.48)	146.49(53.95 to 286.6)	–3.77(–3.96 to –3.58)
Southeast Asia	60 to 64	683.13(433.11 to 961.78)	206.88(77.29 to 407.15)	–3.85(–3.92 to –3.78)
Southeast Asia	65 to 69	814.28(507.93 to 1144.74)	253.67(97.09 to 496.96)	–3.76(–3.89 to –3.63)
Southeast Asia	70 to 74	800.26(496.8 to 1112.32)	263.2(99.4 to 519.75)	–3.58(–3.68 to –3.49)
Southeast Asia	75 plus	566.74(342.33 to 792.69)	204.01(75.39 to 409.22)	–3.29(–3.46 to –3.11)

Figure S1. DALYs of TBLC attributed to APMP and HAP from 1990 t0 2021 across four world regions and five Asia GBD regions.

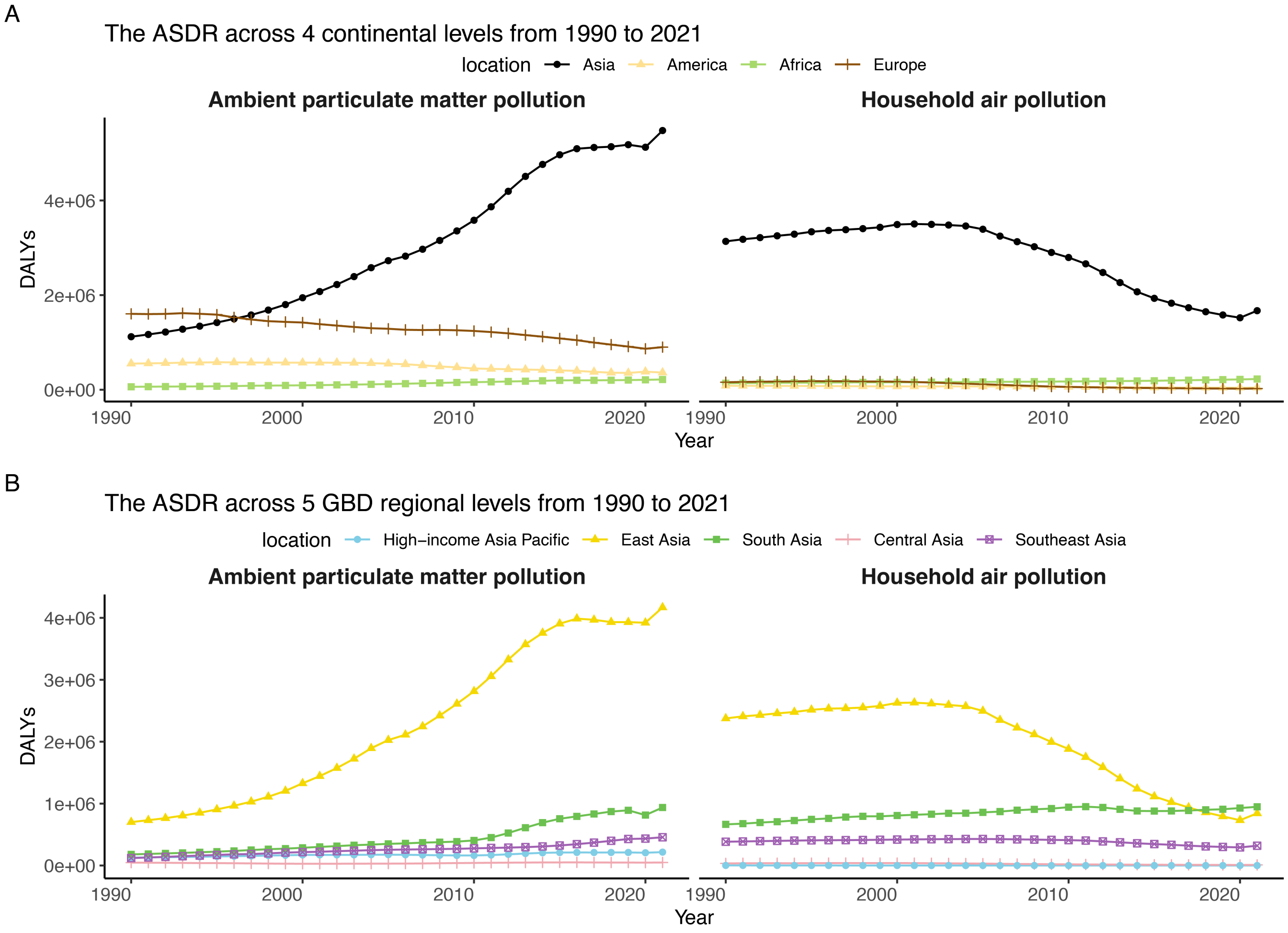

Figure S2. ASDR of TBLC attributed to APMP among 34 Asian countries and territories in 2021.

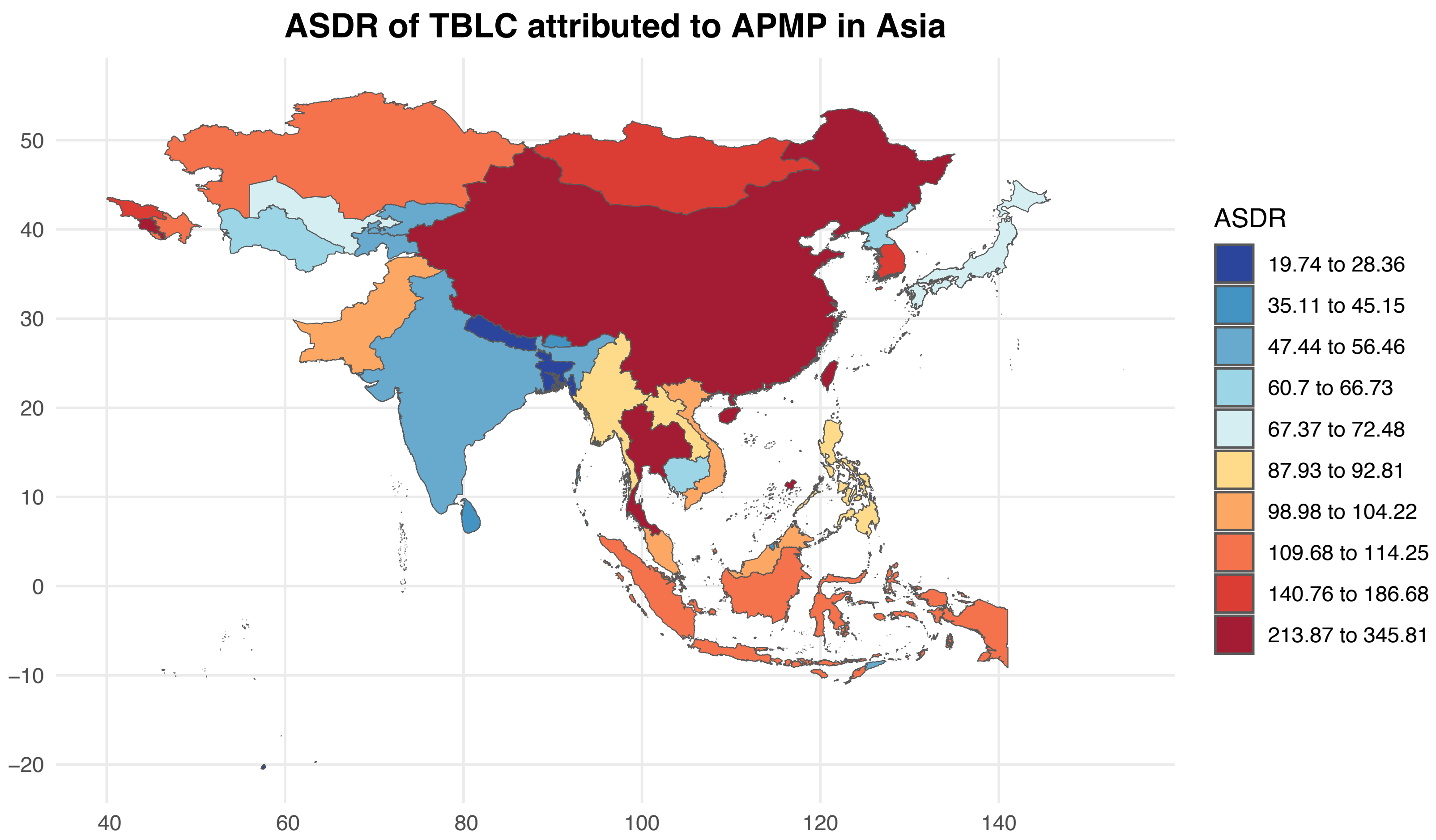

Note: The boundaries on the map are solely utilized to present the distribution of data and should not be interpreted as indicative of administrative borders.

Figure S3. AAPC of ASDR of TBLC attributed to APMP among 34 Asian countries and territories from 1990 to 2021.

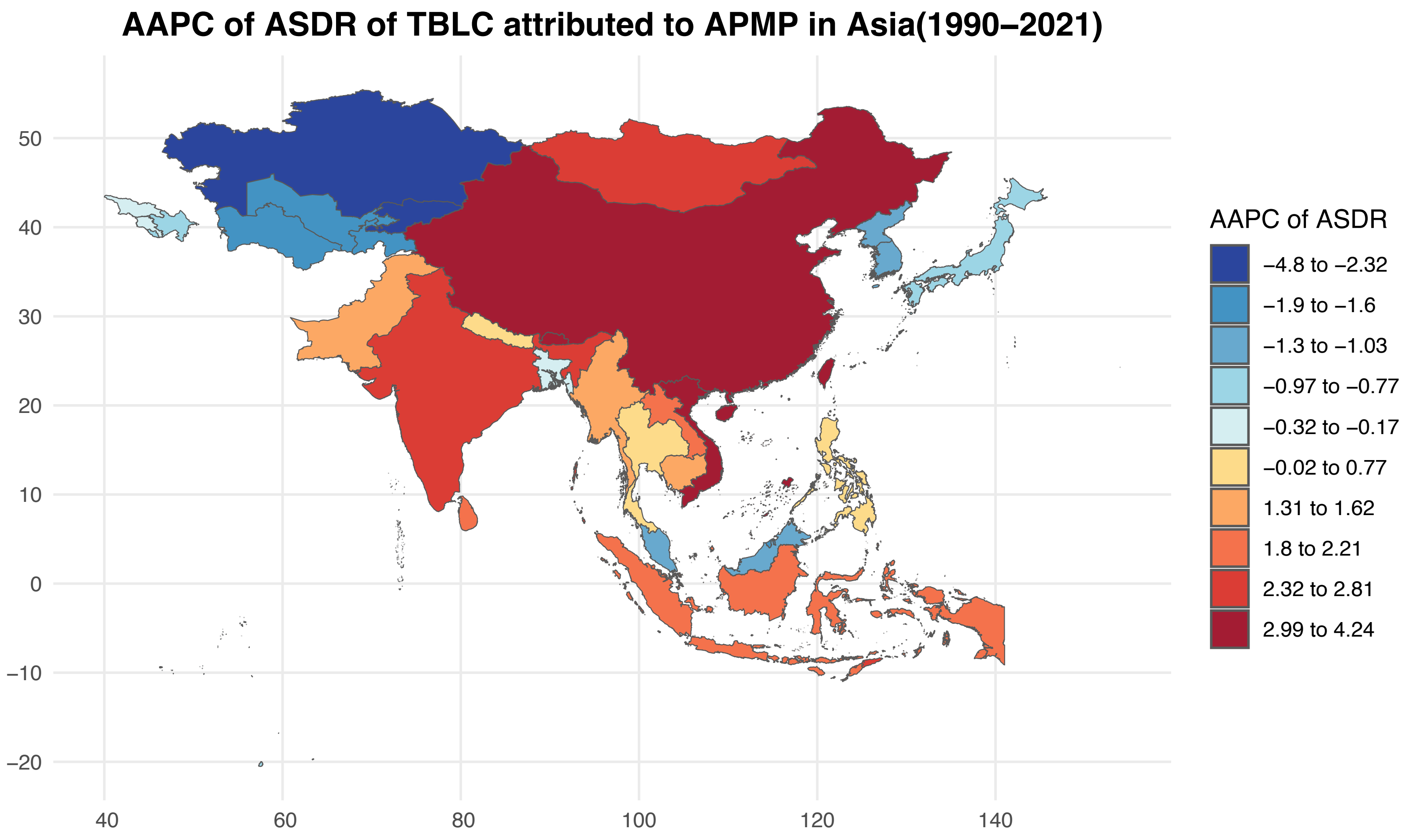

Note: The boundaries on the map are solely utilized to present the distribution of data and should not be interpreted as indicative of administrative borders.

Figure S4. ASDR of TBLC attributed to HAP among 34 Asian countries and territories in 2021.

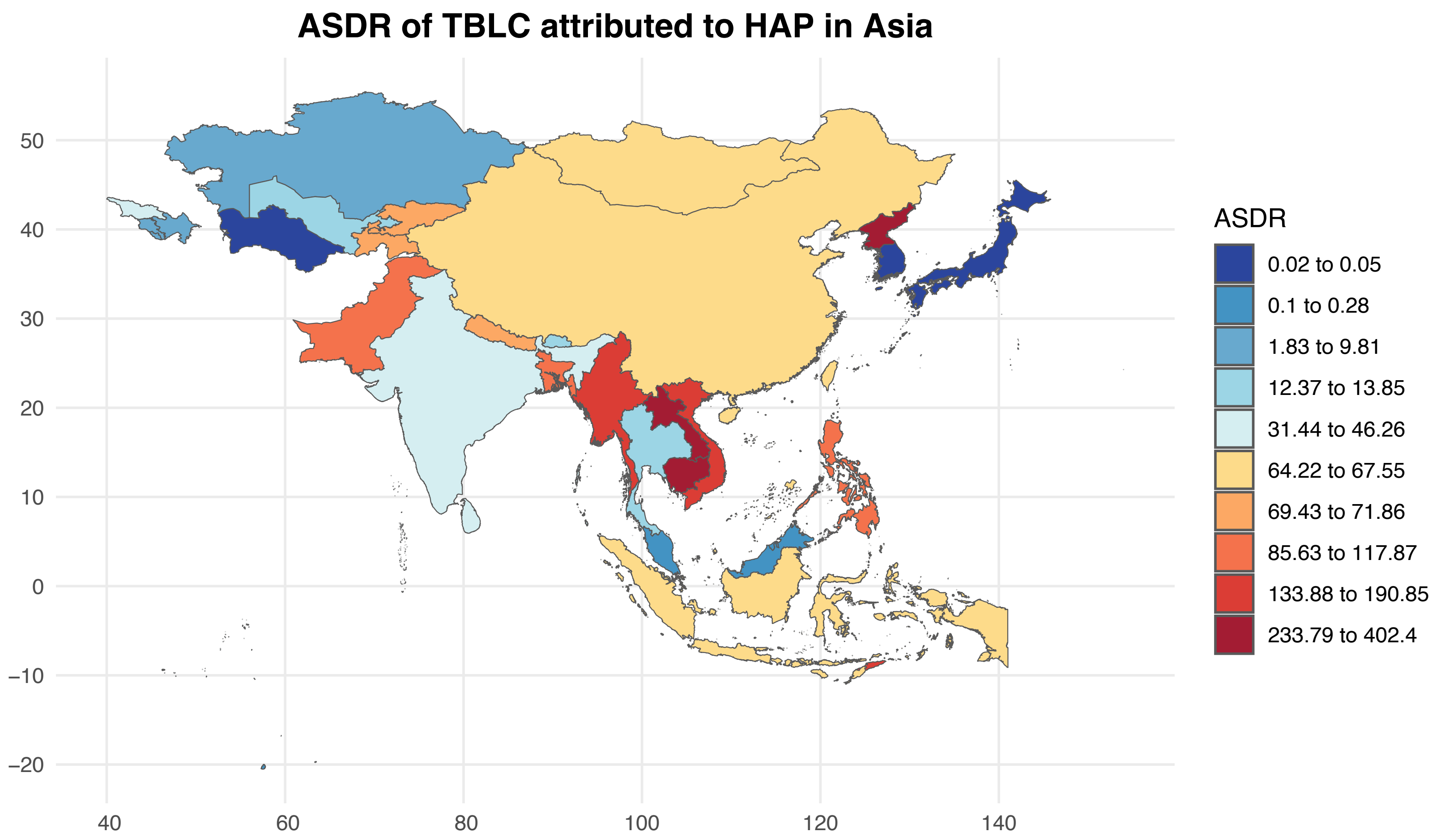

Note: The boundaries on the map are solely utilized to present the distribution of data and should not be interpreted as indicative of administrative borders.

Figure S5. AAPC of ASDR of TBLC attributed to HAP among 34 Asian countries and territories from 1990 to 2021.

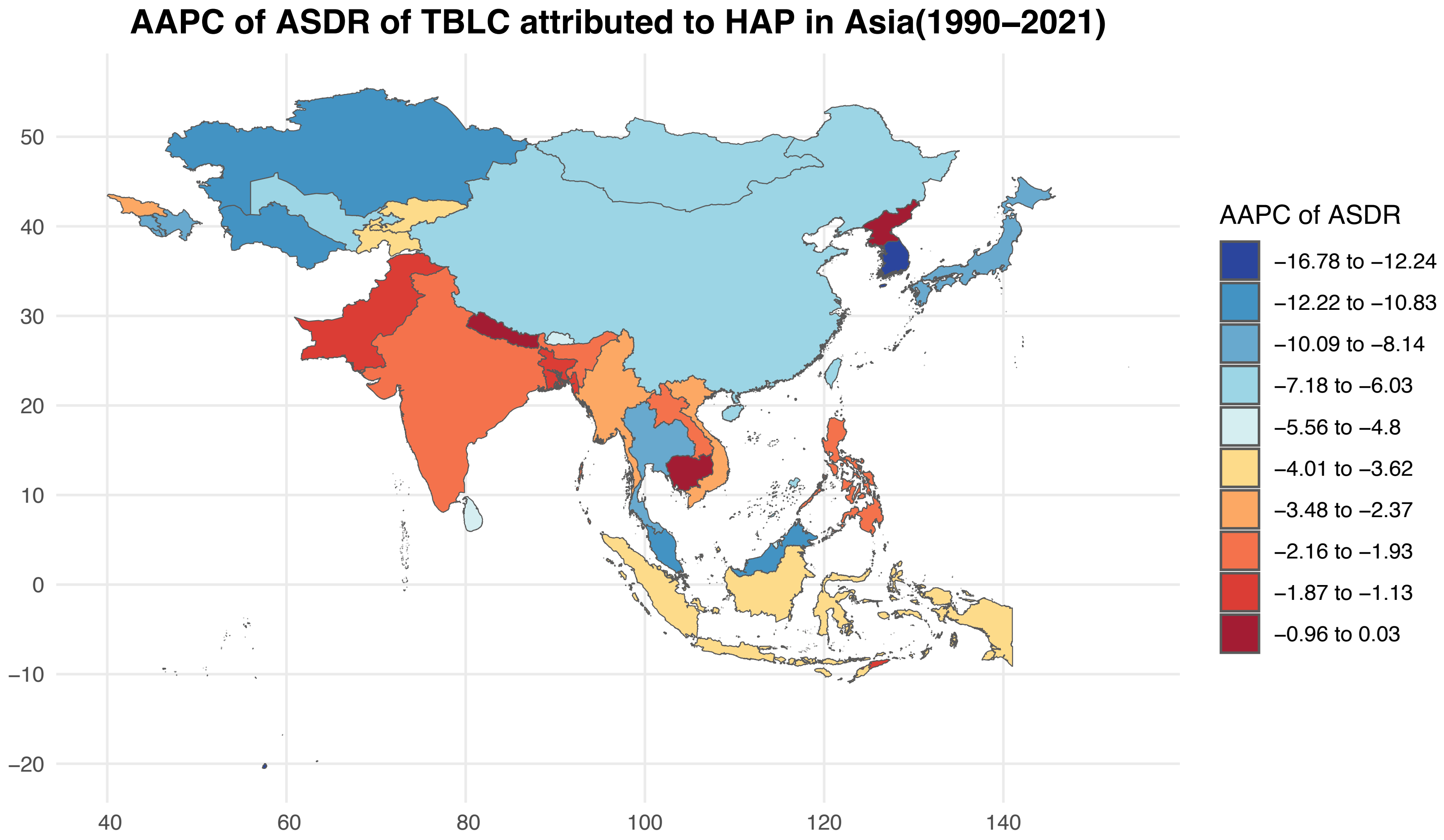

Note: The boundaries on the map are solely utilized to present the distribution of data and should not be interpreted as indicative of administrative borders.
